# Characterisation of white and yellow eye colour mutant strains of house cricket, *Acheta domesticus*

**DOI:** 10.1371/journal.pone.0216281

**Published:** 2019-05-06

**Authors:** Jacek Francikowski, Michał Krzyżowski, Barbara Kochańska, Marta Potrzebska, Bartosz Baran, Łukasz Chajec, Anna Urbisz, Karol Małota, Bartosz Łozowski, Malgorzata Kloc, Jacek Kubiak

**Affiliations:** 1 Department of Animal Physiology and Ecotoxicology, Faculty of Biology and Environmental Protection, University of Silesia in Katowice, Katowice, Poland; 2 The Faculty of Veterinary Medicine, Wroclaw University of Environmental and Life Sciences, Wroclaw, Poland; 3 Department of Embryology and Histology, Faculty of Biology and Environmental Protection, University of Silesia in Katowice, Katowice, Poland; 4 The Houston Methodist Research Institute, Houston,Texas, United States of America; 5 The Houston Methodist Hospital, Department of Surgery, Houston, Texas, United States of America; 6 The University of Texas, M.D. Anderson Cancer Center, Department of Genetics, Houston Texas, United States of America; 7 Department of Regenerative Medicine and Cell Biology, Military Institute of Hygiene and Epidemiology (WIHE), Warsaw, Poland; 8 UnivRennes, CNRS, IGDR (Institute of Genetics and Development of Rennes), UMR 6290, Cell Cycle Group, Faculty of Medicine, Rennes, France; Freie Universitat Berlin Fachbereich Biologie Chemie Pharmazie, GERMANY

## Abstract

Two eye-colour mutant strains, white (W) and yellow (Y) of house cricket *Acheta domesticus* were established in our laboratory. We phenotyped and genotyped the mutants, performed genetic crossings and studied the eye structure and pigment composition using light and electron microscopy and biochemical analysis. We show that W and Y phenotypes are controlled by a single autosomal recessive allele, as both traits are metabolically independent. The analysis of the mutants`eye structure showed a reduced number of dark pigment granules while simultaneously, and an increased amount of light vacuoles in white eye mutants was observed. Significant differences in eye pigment composition between strains were also found. The Y mutant had a lower number of ommochromes, while the W mutant had a lower number of ommochromes and pteridines. This indicates that mutated genes are involved in two different, independent metabolic pathways regulating tryptophan metabolism enzymes, pigment transporter granules or pigment granule formation.

## Introduction

Insect eye-colour mutants are important research models in biological sciences [1–5]. The analysis of eye colour-linked mutations in *D*. *melanogaster* confirmed the chromosomal theory of heredity and for the first time allowed linking a single gene with its locus on the chromosome (the sex chromosome) [6,7]. The majority of studies in this field use holometabolous insect models such as Diptera [8,9], Coleoptera [10], Hymenoptera [11] and Lepidoptera [12,13] while Hemimetabola are often neglected. Within Hemimetabola the Hemiptera order contains the most common hemimetabolous colour mutant groups [14–17]. The genes determine the eye colour traits code for proteins associated with the eye pigmentation and other eye-independent functions. The eye colour mutants are commonly used as the experimental models to study insect genetics [1,16,18,19], body pigmentation [5,20], general physiological processes [2,21–23], behaviour [24,25] and aging [26]. In addition, the eye colour mutants and genes related to this mutation have recently been studied as potential markers for genetic transformation in various insects [10,27].

The colour of insect compound eye is broadly determined by the nature of different pigments [5]. Ommochromes and pteridines are essential pigments that contribute to eye pigmentation [4,5,28–30]. Ommochromes are products of tryptophan metabolism while pteridines are synthesised from guanosine triphosphate (GTP) [5]. A dysfunction of the transporter or the enzymes involved in the synthesis pathway of these pigments leads to aberrant pigment content and results in the modification of the wild type eye colour [10,11,24]. The ommochromes and pteridines are important for eye functionality, reception of visual stimuli [22,31], free radicals scavenging [5,32,33] and functionality of the immune system [34]. Moreover, the synthesis pathways of these pigments are tightly coupled with the metabolism of bioactive compounds or key neurotransmitters such as dopamine and serotonin [5,35]. The guanine derived from GTP and a cofactor of the three aromatic amino acid hydroxylase enzymes, the tetrahydropterin (BH4) are required for the synthesis of dopamine [36], while tryptophan is a precursor of serotonin [37]. Studies showed that *Drosophila melanogaster* eye colour mutants have altered levels and distributions of dopamine and serotonin neurotransmitters [24,37].

The mutations that affect insect eye pigmentation can be divided, depending on the category of pathway they target, into four groups affecting: (1) xanthommatin biosynthesis, (2) pteridine biosynthesis, (3) transmembrane transport of eye pigments and/or pigment precursors, and (4) pigment granule formation [30,38–40]. Several genes regulating the processes listed above had been identified in *D*. *melanogaster* [1]. The transmembrane transport of eye pigments and/or pigment precursors depends on three transporter genes: *white*, *scarlet* and *brown* [40,41]. The formation of the pigment granules, which are the pigment-containing organelles located within the pigment cells, depends on several genes such as *garnet* and *light*, which are involved in the delivery of proteins necessary for granule biogenesis and maintenance [41].

The cricket (Orthoptera, Gryllidae) is the fundamental hemimetabolous insect model for the fields of behavioural research [42] and leg regeneration [43]. A comparison between this hemimetabolous evolutionary older insect species and holometabolous *D*. *melanogaster* can provide valuable comparative information about the metabolism and functions of insects’ eye pigments. It is known that in hemimetabolous insects, such as the Hemiptera, the eye colour mutations are derived from the changes in a single gene [15–17]. So far, only one eye colour mutant has been reported in Orthoptera, in the yellow-eyed *Gryllus bimaculatus* strain from Japan [44]. However there is no information about the gene(s) affected by this mutation [45].

Yellow (Y) and White (W) eye colour strains of the house cricket *Acheta domesticus* derived from the spontaneous mutations were established in our laboratory in Katowice, Poland in 2015. The mutated-eye phenotype is observable in all developmental stages from the nymph to imago. In order to characterise these two mutated strains and make them available for further research, we first determined if these were sex-independent and single gene mutations, and then we investigated in detail the eye pigment granules and pigment content in the mutants.

## Materials and methods

### Establishment of yellow and white eye colour cricket strains

A single yellow-eye (Y) imago male was found by chance in a laboratory culture of wild-type, black-eyed (B) crickets devoted mostly to a behavioural and toxicological genetic research in 2015 at the University of Silesia in Katowice, Faculty of Biology and Environmental Protection, Poland. In order to establish a yellow-eye color colony, the yellow-eye male was paired with the black-eye females. The obtained offspring were all black-eyed. These black-eye progenies (F1) were subsequently interbred and produced both black- and yellow-eye offspring (F2). The yellow-eye females and males were isolated to establish a pure yellow-eyed strain. In the third generation, four white-eye females and three males were found. The yellow (Y, genotype WW/yy) and white (W, genotype ww/yy) eye phenotypes had persisted in the colony for over one year, since 2015. The black-eyed (B, genotype WW/YY) strain used in this study is the original laboratory strain in which no further yellow or white-eye mutants were observed. All strains were reared at 30^0^ ± 2°C and 40% ± 10% relative humidity with a 12:12 light:dark cycle (light source: incandescent lamp Spectrum W0J21508, T8 G13 18W 6500K). During their whole life, the insects had access to shelter, water and food *ad libitum*.

### Genetic crosses

To determine the relationship between genes and heredity, crosses between all strains were conducted. Males and females, 5 days after imaginal molt, were used for the experiment. In each testcross 24 pairs were tested: 12 pairs for males and females (from all strains) in different configurations (yellow male x black female, black male x yellow female etc.). Eye colour was assessed at the 3rd-instar nymph stage in the filial generation. Although the sequencing of the wild type and mutated genes is still in progress and the exact source of phenotype changes remains unknown, for the purpose of this study the observed traits were assigned to the theoretical genes: y—yellow eye gene, and w—white eye gene. For the clarity of the crosses description, the Wild strain (B) was described as WW/YY, the Yellow strain as—WW/yy, and the White strain as—ww/yy.

For backcross procedure, heterozygous individuals (F1) from crosses of BxW (Ww/Yy), BxY (WW/Yy), WxY (Ww/yy) were crossed with recessive homozygote: White (ww/yy) or Yellow (WW/yy) respectively, in both sex configurations. All configurations were made in six replicates. Phenotypes from obtained progeny were counted, and an analysis of the frequency distribution was conducted utilizing a Chi-square test or binominal test.

### Light and transmission electron microscopy (TEM)

Adult specimens (males) of the wild-type and eye colour mutants (20 specimens from each strain) were decapitated. Their head capsules were opened, and the eyes were isolated. The morphology of the eyes was analysed using an Olympus SZX16 stereoscope. A series of images were acquired using an Olympus DP72 digital camera mounted on the stereoscope. For light and TEM microscopy, the eyes were fixed with 2.5% glutaraldehyde in a 0.1 M sodium phosphate buffer (pH 7.4) at 4°C for 24h. After washing in phosphate buffer (5 times, 30 min each), the material was post-fixed in 1% osmium tetroxide in a 0.1 M phosphate buffer (4°C, 2h), rinsed with the same buffer and dehydrated in a graded series of ethanol (30%, 50%, 70%, 90%, 96% and 100%, each for 15 min), acetone (2 times, 15 min each) and then infiltrated successively through mixtures of acetone and Epon 812 resin (3:1, 1:1, and 1:3). Thereafter, the material was embedded in Epon 812 resin (Fullam Inc., Latham, NY, USA) and polymerised into the resin blocks at 60°C for 48 h. The semi- and ultra-thin sections were cut with a diamond knife using a Leica Ultracut UCT25 ultramicrotome. Semi-thin sections (600 nm thick) were stained with 1% methylene blue in 0.5% borax and analysed using an Olympus BX60 light microscope. Ultra-thin sections (70 nm thick) were stained with uranyl acetate and lead citrate and examined with a Hitachi H500 transmission electron microscope. A quantitative analysis of light vacuoles was made on horizontal eye sections (light microscopy) from wild and mutant cricket strains. The comparison of the area was made on sections cut below crystal cone (visible rhabdomeres). The analysis of digital photos was performed with the use of ImageJ software on randomly selected areas (500x500 pix) [46]. Light vacuole areas were quantified as the percentage of selected squares.

### Pigments extraction

To determine the biochemical basis of the yellow and white-eye phenotype, ommochromes and pteridines were extracted using two different methods described by Nijhout (1997) and Tomic-Carruthers et al.(1996) [47]. The 10-day-old imagoes (males) were used to extract the pigments (females were tested in a preliminary study, and there was no difference in pigment content between females and males). For ommochromes and pteridines extraction, 6 pairs of eyes were isolated from insects of each phenotype. For ommochromes extraction, the eyes were homogenised individually in 1% HCl in methanol (150 *μ*L/50mg of tissue) and extracted in the dark for 1 hour. Each homogenate was centrifuged at 10 000 rpm for 10 min. For pteridines extraction, the eyes were homogenised individually in a mixture of 3.5% aqueous ammonia and n-propanol 1:2 (150 *μ*L/50mg of tissue) and extracted in the dark for 1hour. The homogenates were centrifuged at 10 000 rpm for 10 min.

### Thin layer chromatography (TLC)

TLC is one of the most fundamental compound separation methods, often used in insect eye pigment analysis. 5 *μ*L of pigments extract was applied to HPTLC plate (Pre-coated HPTLC plates Nano-Adamant layer 0.2 mm, silica gel 60) under dim light. Extracts from six insects per strain were used in this method. After drying for 10 minutes, the plate was placed in the chromatographic horizontal chamber. Two different mixtures were used as a developing solvent: phenol: water (3:1) for ommochromes, and 1% aqueous ammonia and n-propanol (1:2) for pteridines. The plate was developed for 90 min and dried in the dark for 12 hours. Dry plates were visualised using a UV transiluminator (365 nm) and archived digitally for further analysis. For spots detection, Rf values and areas determination, the digital images were analysed with CpAtlas v. 2.0 [48] software. To determine spots colour, ImageJ software was used for RGB values measurement [49].

### Spectrophotometry

The overall concentration of compounds in the extraction mixture was evaluated by spectrophotometry. Concentration of both ommochromes and pteridines was measured on microplates (UV Cornstar GE half- area 96 wells) using an Infinite M200 TECAN reader. For pteridines, the supernatant was diluted at 1:3 ratio with the extraction solution. Thereafter, the mixture was applied into the wells, and the clear extraction solvent was used as a baseline. The absorbance value was measured at the 230–350 nm (2 nm step) wavelength range. Ommochromes extraction solvents were used undiluted, and the clear extraction solvent was used as a baseline. The absorbance value was measured at the 230–650 nm (2 nm step) wavelength range. For each group of pigments, six replicates per phenotype were used.

### Spectrofluorometry

The fluorescence value of compounds in the extraction mixture was evaluated by spectrofluorometry. The fluorescence of ommochromes and pteridines was measured with a microplate reader (Spark 10M TECAN) on Thermo Fisher Scientific- Nunclon 96 Flat Black microplates. Due to a too high emission, the extraction solution was diluted 40x for pteridines, and 200 μL of mixture was applied into the wells. Ommochromes extraction solution remained undiluted. Clear extraction solvent was used as the baseline. The excitation wavelength was set on 365 nm, and the emission value was quantified at the spectrum window 400–600 nm (excitation/emission bandwidth 20 nm, integration time 250 s, 1 nm step). For each pigment group, six replicates per phenotype were used.

### Statistical analysis

The statistical analysis was performed using Prism 6.0 GraphPad software. The dependence tests were used to compare expected frequencies and the frequencies of traits obtained in genetic crosses. Depending on the number of groups, either a binominal or Chi^2 test was used. A parametric ANOVA test (Tukey test, p<0.05) was used for quantitative analysis of the absorbancy maxima, TLC spots and white vacuoles area.

## Results

### Genetic crosses

The series of crossbreeds between individuals from studied lines provided a complete set of information about the inheritance of the studied traits (Table 1). In heterozygous generation (F1), all individuals exhibited a dominant phenotype, black or yellow; black eyes for B x Y and B x W crosses, and yellow eyes for Y x W. The next generation (F2), derived from crossing heterozygous BY and WY individuals consisted of typical unigenous distribution (3:1) of phenotypes, with the black and yellow dominant phenotype, respectively. A similar distribution was observed independently of sex sets. The more complex picture appeared in the F2 generation after B x W cross. Observed distribution fitted the model 12:3:1 (black: yellow: white). In this model, the offspring containing the recessive homozygotic white gene and heterozygotic yellow gene or dominant homozygotic yellow gene (ww/Yy, ww/YY) expresses black eye phenotype. The results of the B x Y and Y x W crosses indicate that the two observed phenotype traits are determined by two independent genes. There is no evidence that these traits are associated with the sex. This interpretation is supported by the result of the series of backcrosses (Table 2). The results of heterozygotes and recessive homozygotes crosses (Y and W) are similar to the expected distributions (1:1).

**Table 1:** Results of genetic crosses.

generation F1 phenotypes												generation F2 phenotypes												P value	P (two-tailed)
parental phenotypes		number of offspring				observed distribution			theoretical distribution			number of offspring				observed distribution				theoretical distribution			Binominal test (one tailed)		
female	male	B	Y	W	Sum	B	Y	W	B	Y	W	B	Y	W	Sum	B		Y	W	B^a^	Y^b^	W^c^		
*B*	*Y*	967	0	0	967	1	0	0	1	0	0	2966	935	0	3901	3,04	1		0	3	1	0	0,0703	0,1391	ns
*Y*	*B*	1192	0	0	1192	1	0	0	1	0	0	3141	1047	0	4188	3,01	1		0	3	1	0	0,5059	> 0,9999	ns
																								
*Y*	*W*	0	1342	0	1342	0	1	0	0	1	0	0	1376	441	1817	0	3,12		1	0	3	1	0,3109	0,6184	ns
*W*	*Y*	0	1191	0	1191	0	1	0	0	1	0	0	1085	350	1435	0	3,1		1	0	3	1	0,3088	0,6045	ns
																							Chi-square		
*B*	*W*	1054	0	0	1054	1	0	0	1	0	0	2944	698	316	3958	11,9	2,82		1,27	12	3	1	21,86	*	
*W*	*B*	985	0	0	985	1	0	0	1	0	0	3350	727	360	4437	12,08	2,62		1,29	12	3	1	38,04	*	

Eye colour in offspring of intraline, reciprocal or F_1_ crosses between black (B), yellow (Y) and white (W) eye house crickets. Predicted genotypes:

a—WW/YY, Ww/Yy, ww/YY, ww/Yy

b—WW/yy, Ww/yy

c—ww/yy.

**Table 2:** Results of genetic backcrosses.

Parental strains for backcrosses														
Parental phenotypes of heterozygote	Phenotype of recesive homozygote			Number of offspring				Theoretical distribiution			Observed distribiution			P value
F/M		F	M	B	Y	W	Sum	B^a^	Y^b^	W^c^	B	Y	W	Chi2 value
BW	x	W		1936	814	884	3634	2^a^	1^b^	1^c^	2,13	0,90	0,97	18,28
BW	x		W	1336	498	805	2639	2	1	1	2,03	0,75	1,22	71,84
WB	x	W		814	398	420	1632	2	1	1	2,00	0,98	1,03	0,6029
WB	x		W	644	330	316	1290	2	1	1	2,00	1,02	0,98	0,307
														Binominal test (one tailed)
BW	x	Y		1274	1306	0	2580	1^d^	1^e^	0	0,99	1,01	0	0,2708
BW	x		Y	1818	1804	0	3622	1	1	0	1,00	1,00	0	0,4145
WB	x	Y		1552	1586	0	3138	1	1	0	0,99	1,01	0	0,5439
WB	x		Y	1292	1344	0	2636	1	1	0	0,98	1,02	0	0,1603
BY	x	Y		1318	1304	0	2622	1^f^	1^g^	0	1,01	0,99	0	0,4291
BY	x		Y	1190	1206	0	2396	1	1	0	0,99	1,01	0	0,3796
YB	x	Y		828	872	0	1700	1	1	0	0,97	1,03	0	0,1485
YB	x		Y	1056	1118	0	2174	1	1	0	0,97	1,03	0	0,0954
YW	x	W		0	1058	1064	2122	0	1^h^	1^i^	0	1,00	1,00	0,3796
YW	x		W	0	1090	1124	2214	0	1	1	0	0,98	1,02	0,4248
WY	x	W		0	1272	1218	2490	0	1	1	0	1,02	0,98	0,1441
WY	x		W	0	1199	1241	2440	0	1	1	0	0,98	1,02	0,3952

Eye colour in offspring of intraline, reciprocal backcrosses between heterozygotes and recesive homozygotes of house crickets (black (B), yellow (Y) and white (W) eye). Predicted genotypes of the offspring:

a—Ww/Yy, ww/Yy

b—Ww/yy

c—ww/yy

d—WW/Yy, Ww/Yy

e—Ww/yy, WWyy

f—WW/Yy

g -WW/yy

h—Ww/yy

i—ww/yy.

### Structure of the *A*. *domesticus* eyes

**Fig 1:**
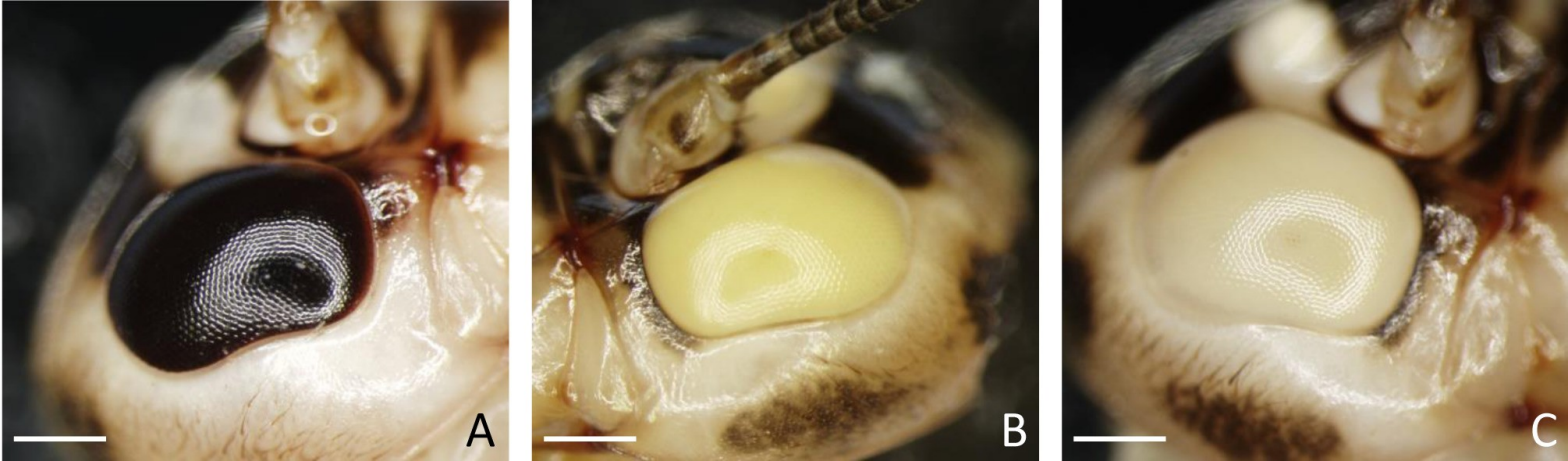
Differences in eye colour of three examined strains. Wild-type black (A), yellow eye mutants (B), white eye mutants (C). Stereo microscope images. Bar is equal to: A. = 0.88 mm, B. = 0.81 mm, C. = 0.82 mm.

**Fig 2:**
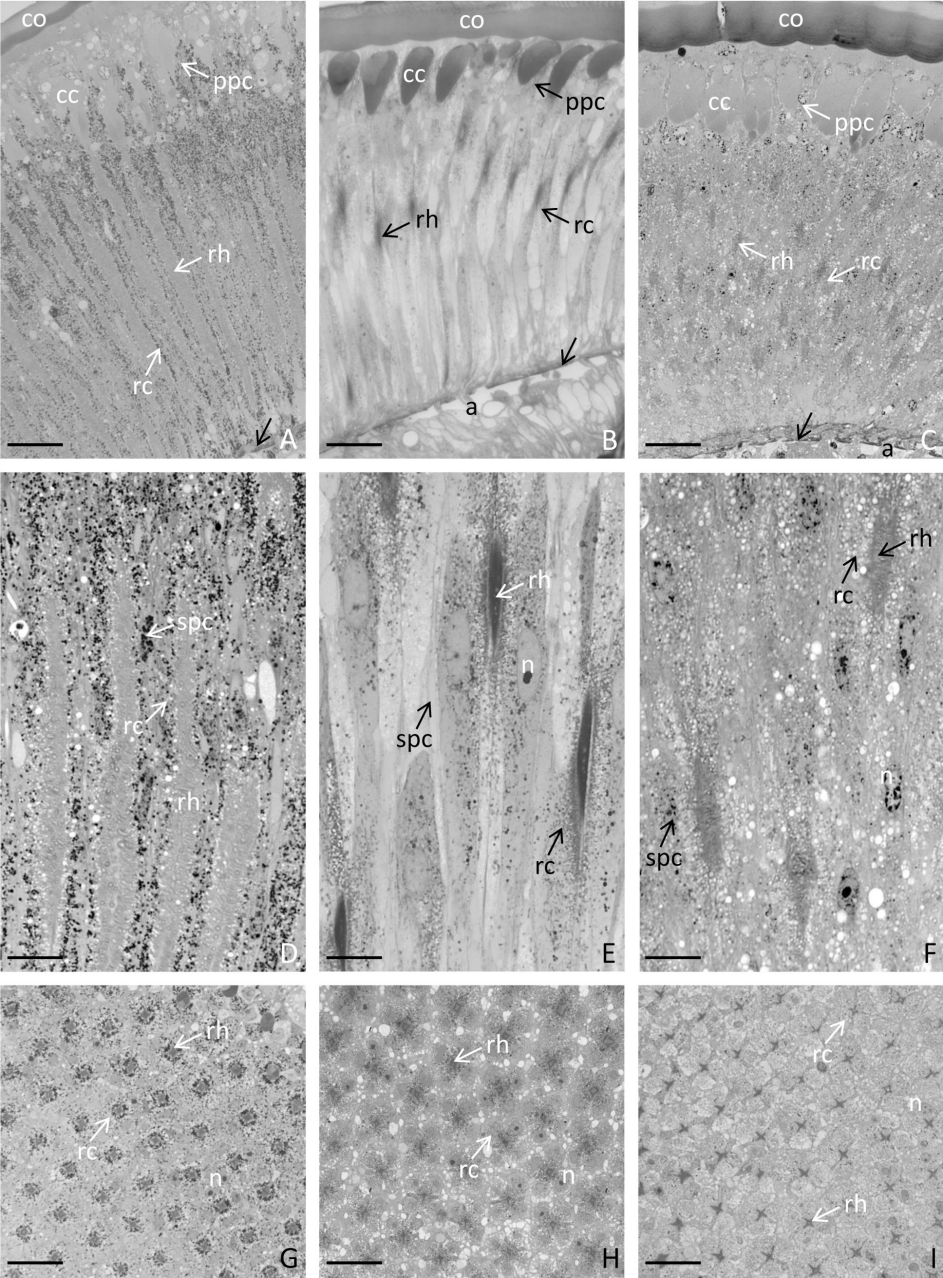
General morphology of the eye of *Acheta domesticus*. The single eye consists of hundreds of ommatidia, which consist of the smooth cornea (co), crystalline cone (cc), primary pigment cells (ppc), retinula cells (rc) and the secondary pigment cells (spc). Lateral membranes of retinula cells form microvilli, which are arranged in the rhabdom (rh). Basement membrane (black arrow), axons of retinula cells (a), nucleus (n). Wild-type black (A, D, G), yellow eye mutants (B, E, H), white eye mutants (C, F, I). Longitudinal sections (A-F), cross sections (G-I). Light microscope. Bar is equal to: D. = 17.12 μm, E. = 18.69 μm, F. = 19.56 μm, G. = 8.56 μm, H. = 9.34 μm, I. = 9.78 μm.

**Fig 3:**
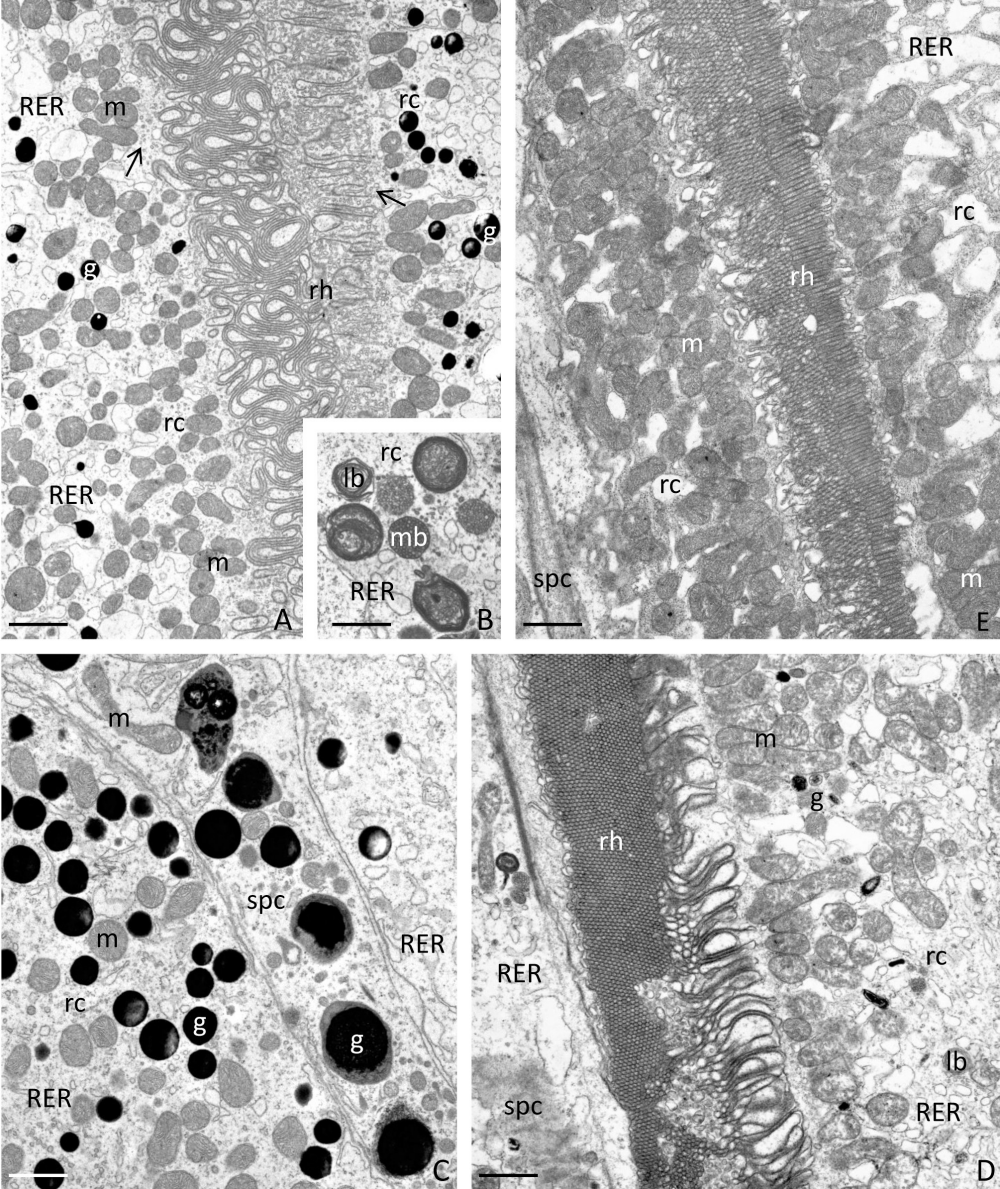
Ultrastructure of retinula cells within the eye of a house cricket. Numerous mitochondria (m), swollen cisterns of rough endoplasmic reticulum (RER), free ribosomes, multivesicular bodies (mb), lamellar bodies (lb) and vesicles with an electron-lucent content (arrows) are present in the cytoplasm of retinula cells (rc). In the wild-type, numerous granules (g) are present. Rhabdom (rh), secondary pigment cells (spc). Wild-type (A-C), yellow eye mutants (D), white eye mutants (E). TEM, bar is equal to: A. = 1.71 μm, B. = 1.29 μm, C. = 0.91 μm, D. = 1.52 μm, E. = 1.52 μm.

**Fig 4:**
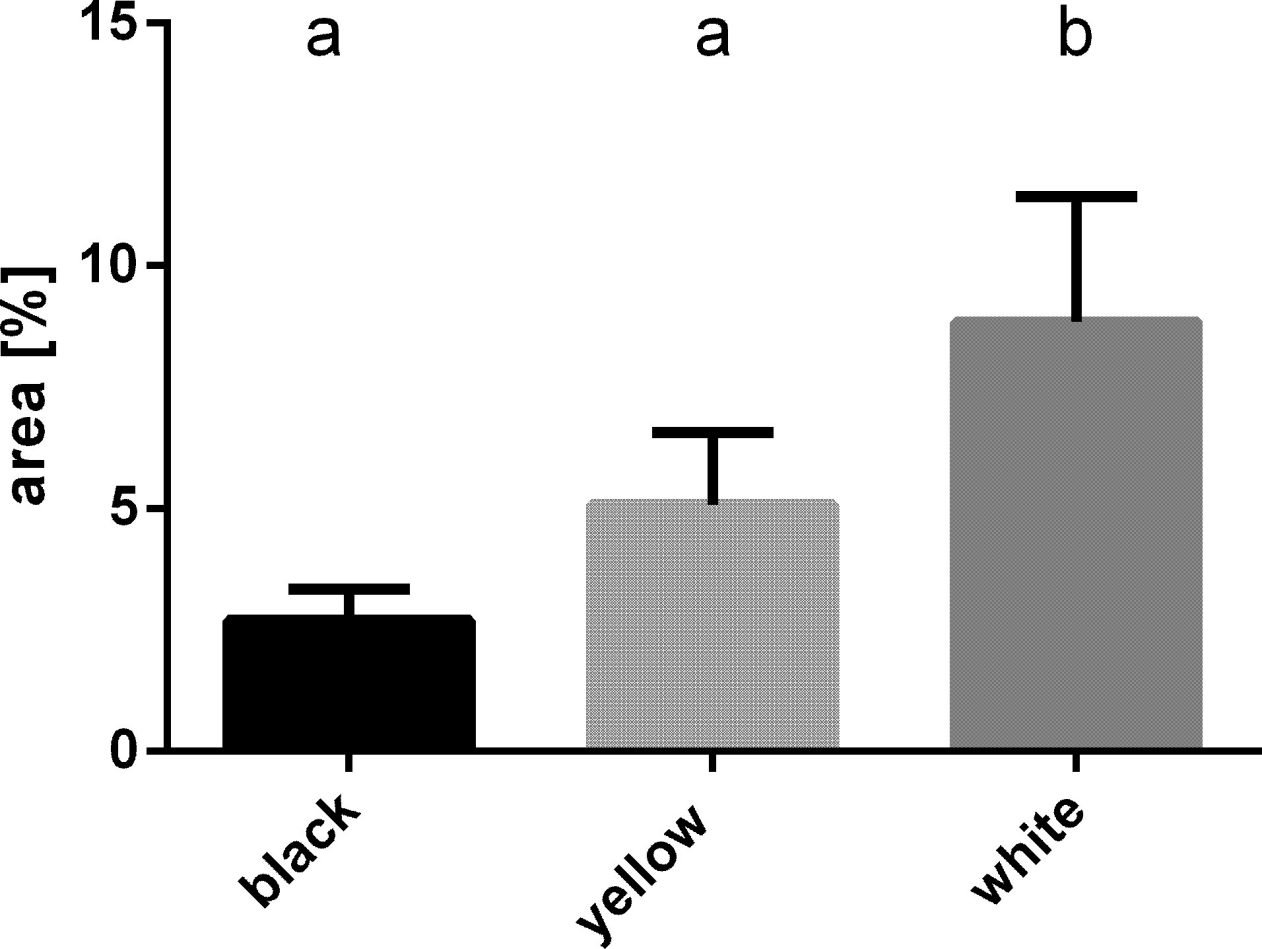
Comparison of the area occupied by light vacuoles in horizontal eye sections from all cricket strains. ANOVA test, p<0.05. Different letters indicate statistically different groups.

Each examined strain (wild type and mutants) had a characteristic, distinctive eye colour. The wild-type had black coloured eyes (Fig 1A), while the mutants had yellow (Fig 1B) or white eyes (Fig 1C). The single eye is hemispherical and consists of hundreds of hexagonal ommatidia (Fig 1A–1C), which rest on a basement membrane (Fig 2A–2C). In both the wild-type and the mutants, the spatial organization of the ommatidia was regular, and they were arranged parallel to each other (Fig 2A–2I). A single ommatidium is composed of a smooth cornea, a crystalline cone surrounded by primary pigment cells, a cluster of the retinula, and the secondary pigment cells (Fig 2A–2I). Lateral membranes of retinula cells form finger-like microvilli, which are arranged in rhabdom in the central part of the ommatidium (Figs 2A–2I, 3A, 3D and 3E). In the basal part of the eye, the axons of retinula cells of each ommatidium exit the eye through the holes in the basement membrane (Fig 2B and 2C). The cytoplasm of retinula cells is rich in mitochondria, swollen cisterns of rough endoplasmic reticulum [50], free ribosomes, multivesicular bodies, lamellar bodies, vesicles with an electron-lucent content (Fig 3A–3E) and light vacuoles (Fig 2D–2F). Clusters of mitochondria accumulate in the vicinity of rhabdom membranes (Fig 3A, 3D and 3E). In the wild type eye, the cytoplasm of retinula cells contained multiple granules with homogeneous or heterogeneous electron-dense content (Fig 3A and 3C). On the semi- and ultrathin sections of the yellow eye mutant, the cytoplasm of retinula cells contained a small number of fine, electron-dense granules (Fig 3D), while in the white-eyed mutants, such granules were never observed (Fig 3E). Furthermore, in the wild type eye, many electron-dense granules were found in the cytoplasm of pigment cells lining the outside of each ommatidium. These granules were not noticed in both yellow and white mutant pigment cells (Figs 2A–2I, 3D and 3E). Many light (low electron density) areas, inside and outside of cells, are visible in horizontal and longitudinal sections of ommatidia of the white strain crickets. The analysis of the region occupied by light vacuoles in horizontal eye sections from all the strains was conducted. Obtained results confirmed significantly higher occurrence and a greater surface occupied by light areas on the eye sections from the white strain in comparison to the yellow and black strains (Fig 4).

### Composition of eye pigments in wild type and mutant eyes

**Fig 5:**
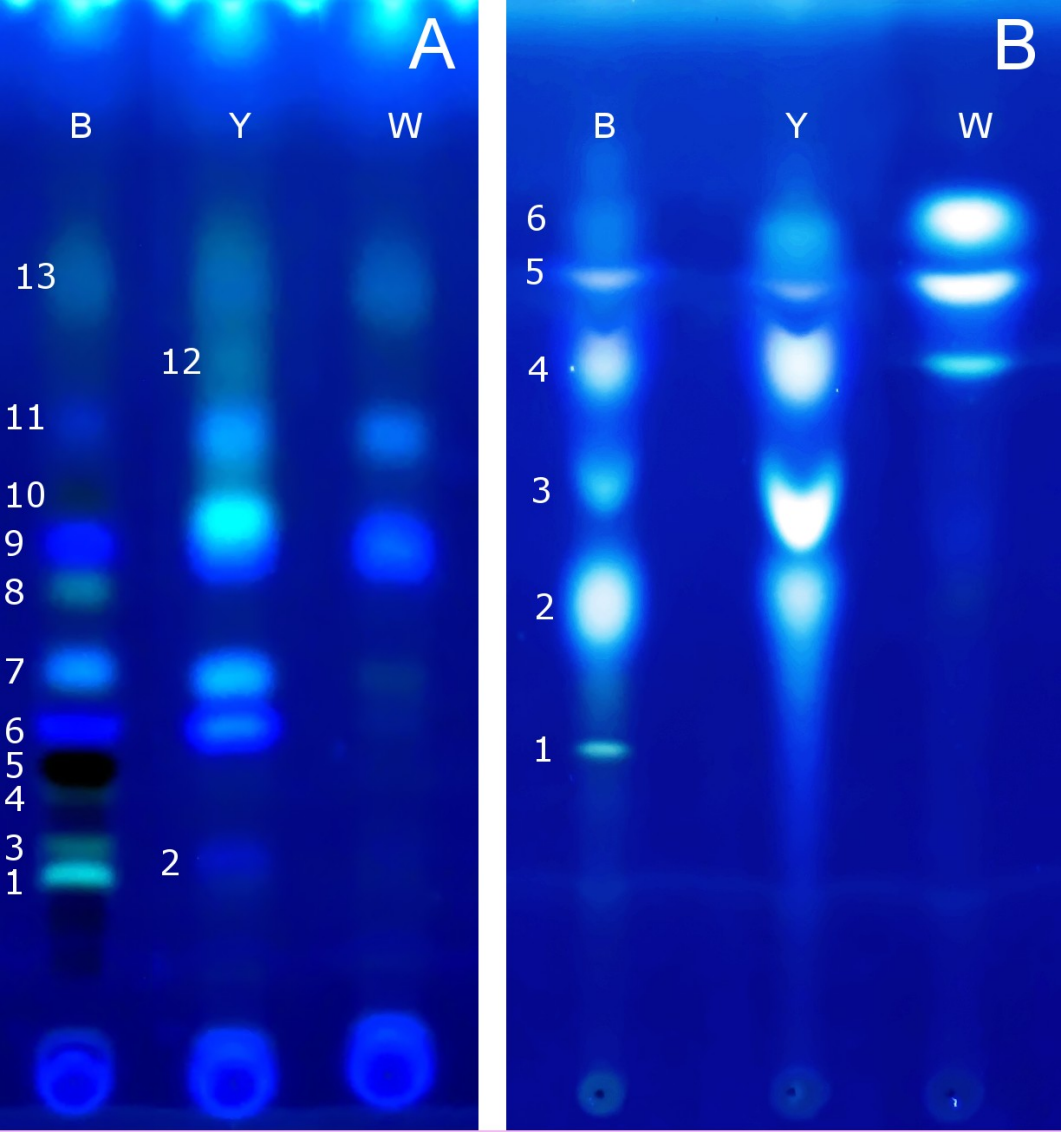
Thin layer chromatography of eye pigments. Image of TLC plates in UV light (345 nm) with profiles of isolated compounds: A) ommochromes B) pteridines for all three tested strain mutants: black- (B), yellow- (Y) and white- (W) eye. Numbers correspond to spots numbers in Table 3.

The TLC analysis of pigment composition was based on the colour and retardation factor (R_f_) of the obtained spots. The results of isolation and separation of pigments from each strain were digitally captured (Fig 5). Although it was not possible to accurately identify most of the isolated compounds (Table 3), the TLC analysis showed that the major changes in the mutant strains involved the ommochromes pigments. In the chromatogram of the black strain, three brown spots were observed with a dominant, dark 5th spot (xanthomatine) which is the main, dark color visual pigment in insects. The major phenotypic difference between wild type and mutant cricket strains was the absence of this brown pigment. The yellow-eyed strain lac the brown pigment (which in the wild type obscures light-coloured pigments), while the white-eyed crickets lacks all eye pigments. In the case of the yellow-eyed crickets, it is hard to tell if the yellow colour is caused by the ommochrome or pteridine pigments because both these pigments cause yellow colouration of the tissue.

**Table 3:** Results of TLC analysis with calculation of Rf values and spot areas for A) pteridines and B) ommochromes. Different letters indicate statistically different groups, ANOVA Tukey test p<0.05.

A)			**B**	**Y**	**W**
colour	spot nr.	rf	mean area ± SD		
Mint	1.	0.308	656.8 ± 87.3		
light blue	2.	0.436	7401.4 ± 1003.7 a	5299.8 ± 724.6 b	
summer sky	3.	0.543	1696.5 ± 327.8 a	6660.8 ± 356.1 b	
pattens blue	4.	0.647	3439.4 ± 759.3 a	4999.4 ± 439.8 b	1671.4 ± 235.7 c
light blue	5.	0.728	1285.1 ± 199,2 a	619.4 ± 287.9 b	4090.6 ± 1021.3 c
dodger blue	6.	0.777	1001.9 ± 173.2 a	595.7 ± 211.5 b	6608.3 ± 1105.6 c
B)			**B**	**Y**	**W**
colour	spot nr.	rf	mean area ± SD		
turquoise	1.	0.189	1485.6 ± 259.8		
Blue	2.			1594.5 ± 302.4	
Teal	3.	0.209	197.2 ± 37.6		
dark blue	4.	0.248	655.7 ±116.2		
Black	5.	0.289	2737.4 ± 146.3		
Blue	6.	0.323	3045.5 ± 591.6 a	5127.2 ± 726.9 b	
dodger blue	7.	0.38	2849.1 ± 231.7 a	4083.8 ± 592.4 a	
cerulean	8.	0.46	419.8 ± 87.7		
light blue	9.	0.493	4192.4 ± 878.9 a	7757.7 ± 453.2 b	6074.6 ± 907.5 b
sapphire	10.	0.52	163.4 ± 45.7		
Blue	11.	0.608	1366.8 ± 112.5 a	3258.3 ± 293.4 b	2512.4 ± 198.9 ab
cerulean	12.	0.66		120.1 ± 26.2	
cobalt	13.	0.727	840.6 ± 389.6 a	789.4 ± 187.4 a	1567.7 ± 392.1 b

In summary, the yellow eye strain lacks 6 out of 11 spots present in the black eye strain. Moreover, in the samples from the yellow eye insects, there is one additional pigment spot (designated as # 10) not observed in the other strains. In the white strain insects, only 2 spots out of 11 spots present in the wild strain are observed.

TLC analysis of the pteridines showed the differences in the qualitative and quantitative compounds composition between the wild type and mutant strains. The chromatogram showed the presence of six spots in the wild type strain and only five spots in the yellow eye strain. The white eye strain had only half of the spots present in the wild type strain. It is possible that the lack of the yellow colour in the white strain is associated with the lack of ommochromes and pteridines.

### Spectrophotometric measurement of eye pigments in wild type and mutant eyes

**Fig 6:**
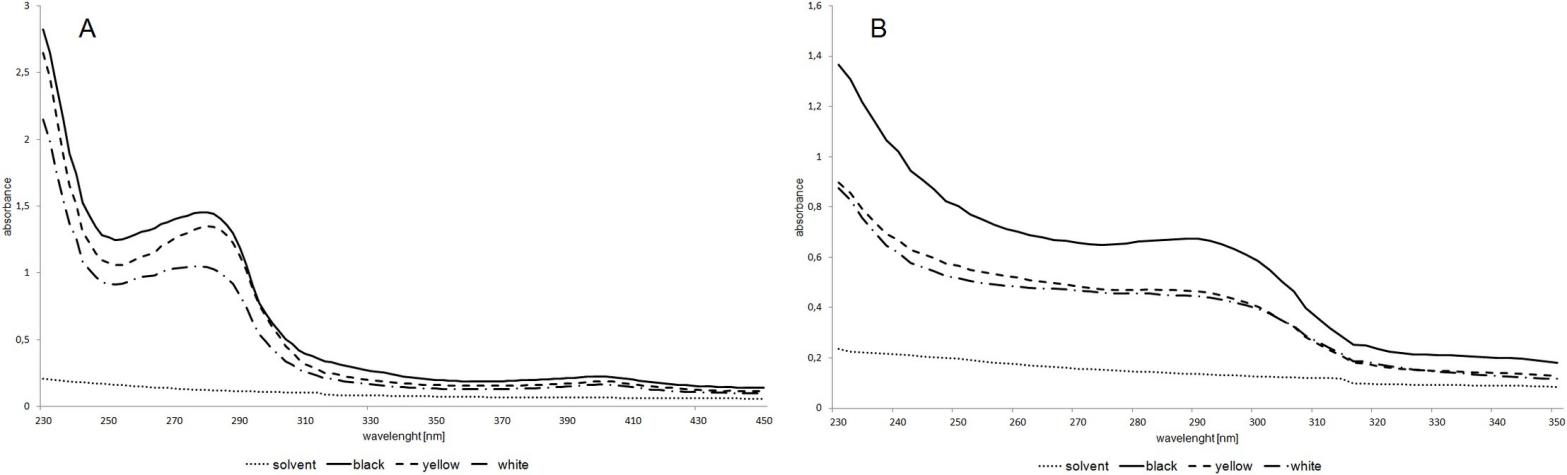
**Spectrophotometry of ommochromes (A) and pteridines (B).** Differences in the absorbency of pigments isolated from the eyes of black-, yellow- and white- eye mutant strains of house crickets.

Spectrophotometry analysis of absorbance pattern of the ommochromes solution showed two distinct peaks of absorbance: a large peak at 280 nm and a much smaller peak at 400 nm. For both of these peaks, the absorbency values are lower for the mutant strains than for the black strain. These results correspond with thin layer chromatography results: more pigments bands are present in the eyes of black crickets than in the mutant eyes. (Fig 6A).

For pteridines, only one absorbance peak was observed. In the black strain, there is an evident absorbance peak at 292 nm. The black strain exhibited a two-fold higher absorbance than the yellow and white strains, which were both similar to each other. This result does not correspond with the results of the TLC assay and suggests the presence of other compounds undetectable by the TLC assay (Fig 6B).

**Fig 7:**
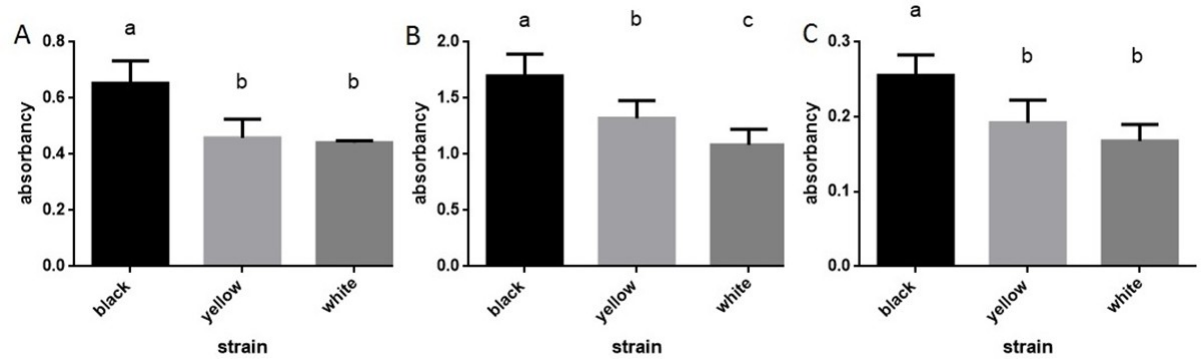
**Mean absorbency (±SD) for pteridines at 280 nm (A), ommochromes at 280 nm (B) and 400 nm (C)**. ANOVA test, p<0.05. Different letters indicate statistically different groups.

Statistical analysis reveals quantitative differences between mutant strains and the wild type in pteridines for the absorbance peak value - 280nm. There was no difference between mutant strains (Fig 7A). For ommochromes, the quantitative analysis for two visible peaks (280 and 400 nm) was conducted. In both cases, the differences between mutants and the wild type were observed. Significant differences between mutants were observed only at 280 nm (Fig 7B and 7C).

### Spectroflourometric measurement of eye pigments in wild type and mutant eyes

**Fig 8:**
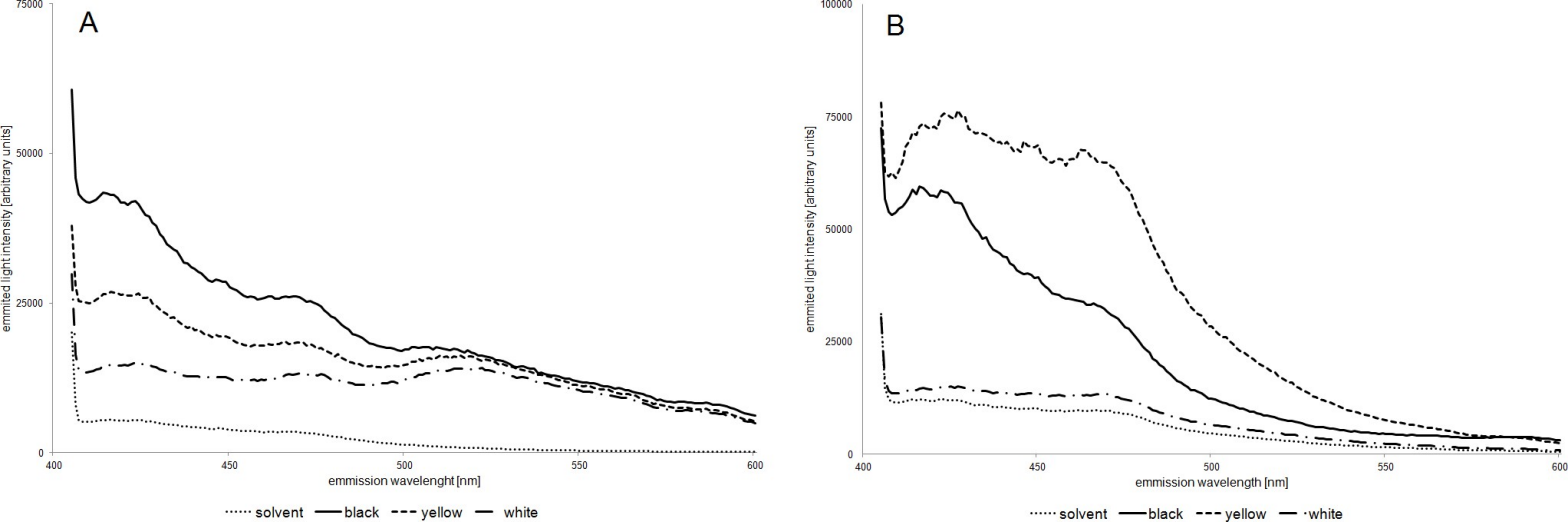
**Spectrofluorometry of ommochromes (A) and pteridines (B).** Differences in the fluorescence of pigments isolated from the eyes of black-, yellow- and white- eye mutant strains of house crickets.

In the fluorometry method only the fluorescent compounds give a positive signal. So, it is possible to see which spots on the TCL plate are the fluorescent compounds. We used fluorometry to quantitate fluorescent compounds in the wild type and mutant strains. In both pigment (pteridines and ommochromes) groups, the baseline fluorescence (solvent fluorescence) was low and stable. We found that the different strains differed in the quantity of ommochromes. The most profound difference was observable at the 410–490 nm spectrum range. There was a strong reduction of overall signal in the yellow and white strain, which corresponded to the reduced number of spots on the TLC plate. The highest fluorescence was observed in the black strain, lower in the yellow strain and the lowest in the white strain. Fluorescent emission in the white strain was residual and close to the background emission. When samples were excited with 495 nm wavelength, the white and yellow emission curves overlapped and the signal had similar intensity (Fig 8A).

The fluorescence measurement of pteridines also revealed differences between strains. Extracts from the eyes of the yellow strain exhibited much higher fluorescence than that of the black strain and also had a different curve shape (Fig 8B). This result can be interpreted as a sign of physiological adaptation and is observed in the wavelength range 435–475 nm; probably the absence of the brown pigments in the yellow eye caused more intense synthesis of pteridines to protect the eyes against light. Moreover, the white strain exhibited the lowest signal intensity, close to the background. This result confirms reduced content of pteridines in the white eye visible on the TLC plate (Fig 5).

## Discussion

In this study we would like to present the characteristic of yellow and white eye colour mutant strains of house cricket (*Acheta domesticus*). Our results provide new information about the novel eye colour mutation in hemimetabolous insects from the Orthoptera order. Previous reports focused either on holometabolous insects [5] or presented only a general overview of the single gene mutations for hemimetabola [15,17,51]. Our results are in agreement with the data from *D*. *melanogaster* eye colour mutants where the content of pteridines and ommochromes is altered [24].

### Genetics

The eye colour mutant individuals were easily distinguishable starting from the first-instar nymph stage of development. The crossing experiments demonstrated that the yellow and white eye phenotypes were inherited as a simple Mendelian autosomal recessive trait. The Mendelian ratio of 3:1 observed for BB x YY, YY x WW crosses and 12:3:1 for BB x WW crosses, and Mendelian ratio 1:1 in backcrosses of BY x YY and YW x WW confirmed a single gene nature of both traits. The lack of changes in the distribution, regardless of multiple replications and sex setups, clearly ruled out that the traits are sex-related. Similar conclusions about mutations and sex relation are also true for other hemimetabolous insects [15,17,52]. Only in one species of Hemiptera, the eye colour mutation was sex-linked [16] contrary to *D*. *melanogaster* where the eye colour mutations are mainly sex-linked [53].

The obtained phenotypes and results of BWxBW crosses close to 12:3:1 indicated that the insects with the genotype ww/YY, ww/Yy have a black eye phenotype. This also indicates that the black eye trait (the presence of dark brown pigments) is connected with the yellow gene and masks the white eye phenotype. The white eye phenotype requires the mutation in both genes. The lack of the pleiotropy effect indicates a genetic and metabolic independence of both mutated genes. It is difficult to compare these results to other studies on similar subjects because they usually describe single changes in the phenotype [54]. Further analysis is necessary to better understand the genetic basis of the observed mutations.

### Eye structure

The development and the structure of the insect eye are determined by a number of biochemical and genetic factors [5,55,56]. Although the genes regulating eye development and structure are well known, there is no information on the relationship between these genes, the eye colour and the process of pigment granules formation. Changes were described in other holometabolous insects [23,50]. So far, there are only two reports describing spontaneous eye colour mutation in the holometabolous insect (in the honey bee *Apis melifera*), which affects the structure of the ommatidia; the *limao* mutant (yellow eyes) [50] *Chartreuse red* (reddish-brown eyes) mutant, and *laranja* (orange eyes) mutant [57]. The analysis of the eye structure in *D*. *melanogaster* eye-color mutants (white, scarlet, vermilion, brown) did not show changes in the ommatidia arrangement or ultrastructure [24,58,59]. There are reports about *D*. *melanogaster* retina degeneration due to the effect of constant light exposure [60–63]. Described modifications became aggravated with age. Our study showed that the mutant strains of crickets have no profound changes in the eye structure. Only the presence of numerous big white vacuoles (presumptive autophagosomes) in the white eyes might indicate some differences; probably a massive autophagy process within the cells [57]. Simultaneously there are some big white spaces between ommatidia, probably lacunae, like in *Drosophila* white mutant eyes [60]. These lacunae may represent the first signs of retinal degeneration. It can suggest some ommatidia degradation processes. In light of these observations it will be necessary to analyse the imago eye structure during ageing to determine the dynamic of the potential changes with time. Cricket mutants from our breeding room had constant access to shelter so they can behaviourally regulate light exposure. It would be interesting to check if this factor can increase eye degeneration in crickets.

In the light of the discussed results, the presented mutants can be a source of new information about genes, proteins and processes related to the development and function of the compound eye in insects. They may also help to link the role of granules and their pigments to the development and metabolism of the eye, as this aspect is generally overlooked. It will also allow assessing the influence of the composition of the pigments on physiology and perceptions in eye colour mutants.

### Eye pigments

There is a general belief that the colour of insect eyes is mainly determined by the type of ommochromes [64–66]. However, many studies indicate that eye pigmentation is a cumulative effect of the interplay between various ommochrome and pteridine pigments [4,41,67]. We show here that the wild-type eye contains both types of pigments. We also show that the white eye mutants have reduced amounts of pteridines and ommochromes in comparison to the wild-type. Consistent with lower pigment levels in the mutants, there is a reduction in the number of pigment granules in the yellow eye strain, and a complete absence of granules in the white eye strain. However, it is still unknown if the lower amount of pigments leads to a reduced number of pigment granules or *vice versa*.

All three methods of analysis applied here showed that the content and composition of the ommochromes differs between cricket strains. There is a significant difference in the quantity of eye pigments and the identity of pteridines and ommochrome pigments. Especially, the lack of brown pigments, i.e. xanthomatin, which give the cricket eyes a distinctive dark brown colour, seems important [68]. The yellow eye phenotype results from the lack of xanthommatin, which masks yellow pigment (xanthopterin) in the wild type eye. In the white eye all the colour pigments (in visible light) are absent. What is interesting is the effect of much higher fluorescence of pteridines extracted from the yellow mutant eye than that of the black strain (Fig 8B) the same as bigger areas of some spots on TLC plates (Table 3). The described effect is especially strong in pteridines from both mutants strains. This result can be interpreted as physiological adaptation. Probably the absence of the brown pigments in the yellow eye caused more intense synthesis of pteridines to protect eyes against light. The second possible explanation is an accumulation of intermediate pigments as the effect of the enzyme mutation. There is no information about similar effects in literature. This, together with the lack of pteridine pigments, suggest the relationship of the mutated gene with both synthesis pathways of ommochromes and pteridines at the substrate level [24,35].

The results regarding the content of pigments obtained using the described methods indicate their unevenness with respect to each other. In several articles, the quantitative analysis of the content of pigments in extracts is carried out using only spectrophotometry [52,69]. Our results obtained from pteridine samples clearly indicate differences between methods, especially in the comparisons between the strains. In the case of the yellow strain, the accumulation of pigments can be seen in fluorometry, while similar results cannot be obtained using spectrophotometry (absorbance). Similarly, for the white strain, fluorometry shows a much lower content of pigments than spectrophotometry. Therefore, we believe that to compare the content of fluorescent pigments in extracts, spectrofluorometry or TLC is more suitable than spectrophotometry. We also believe that for a more complete picture, at least two out of three methods should be used in such analyses. It also seems crucial to measure the whole fluorimetric spectra and not only report the single peak values, which gives a fuller picture of the sample pigment composition [15,17].

There is a considerable number of genes and proteins related to the eye pigments synthesis pathways. The three main genes associated with the changes in eye colour, localised in the fruit fly genome, are white, brown and cardinal. Proteins encoded by the white gene form a transporter complex with the proteins encoded by the other two genes, respectively for the tryptophan and guanine metabolites [5]. However, current knowledge in this area points to numerous genes associated with these pathways, the mutation of which can lead to colour changes in the insects`eyes. It is difficult to precisely place the eye colour mutation within those pathways. Moreover, due to the evolutionary divergence of homo- and holometabolous insects, there are unquestionably significant differences in their ontogenesis and physiology. The comparison of the mRNA sequence of selected genes, such as *white*, *brown*, *vermilion*, *cinnabar*, *cardinal*, *scarlet* from *Gryllus bimaculatus* (Asgard http://asgard.rc.fas.harvard.edu date of access: 08.02.2018) and *D*. *melanogaster* revealed a low sequence similarity between these orthologues. This suggests a low degree of similarity in eye pigments profiles between holo—and hemimetabolous insects.

*D*. *melanogaster* is a model insect whose genes, enzymes and molecules involved in the eye colour phenotype are well known. Much less is known about eye-colour mutants in other than fruit fly insects. Even less is known about the relationship of tryptophan and guanine metabolism pathways and their intermediate metabolites with insect physiology and behaviour [10,66,70–72]. The available data show that insects with disrupted tryptophan pathway, thus visual mutants, may be models for mammalian diseases and pathophysiology such as autism, diabetes, Parkinson’s, and Alzheimer’s diseases [1]. Compounds like kynurenine or biopterin are involved in pathophysiological processes [73–75]. Thanks to these and similar discoveries, a cricket may become a useful model used in future genetic and physiological research. Because of its relatively large size and hemimetabolous type of development, the cricket may become an informative alternative to the fruit fly model [45].

## Supporting information

S1 File Photos of horizontal eye sections for light vacuoles area analysis from all tested strains. (ZIP)

S2 File Spectrophotometry and fluorometry results for pteridines and ommochromes extracted from mutants and wild strain. (ZIP)
