# Supplementary figures and images for: Characterisation of white and yellow eye colour mutant strains of house cricket, Acheta domesticus
Source: PLoS One. 2019 May 6;14(5):e0216281. doi: 10.1371/journal.pone.0216281 (PMC6502451; doi:10.1371/journal.pone.0216281)

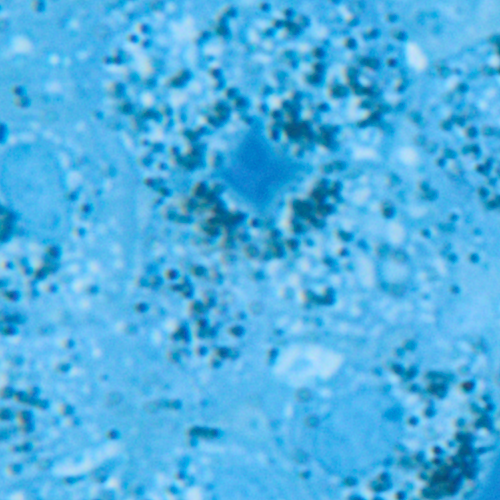

Supplement: S1 File — (ZIP) [file pone.0216281.s001.zip › vacuolas - horisontal eye section photos/black/B_100x_1-1.tif]

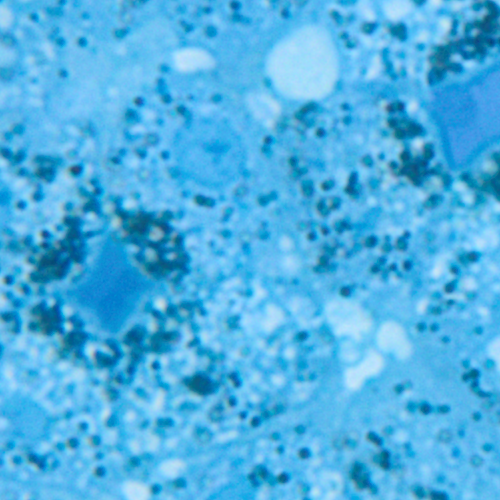

Supplement: S1 File — (ZIP) [file pone.0216281.s001.zip › vacuolas - horisontal eye section photos/black/B_100x_1-2.tif]

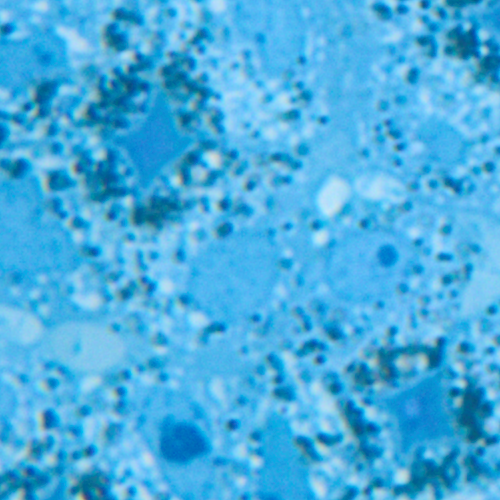

Supplement: S1 File — (ZIP) [file pone.0216281.s001.zip › vacuolas - horisontal eye section photos/black/B_100x_1-3.tif]

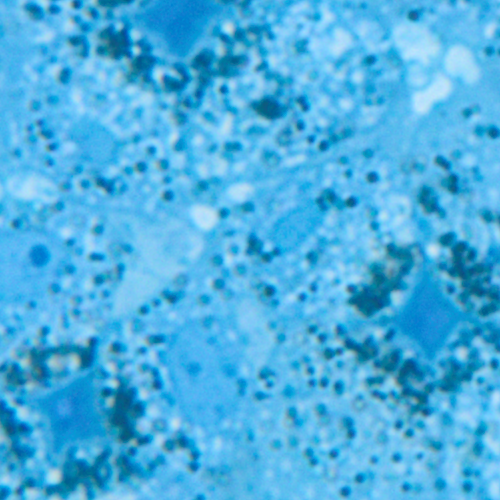

Supplement: S1 File — (ZIP) [file pone.0216281.s001.zip › vacuolas - horisontal eye section photos/black/B_100x_1-4.tif]

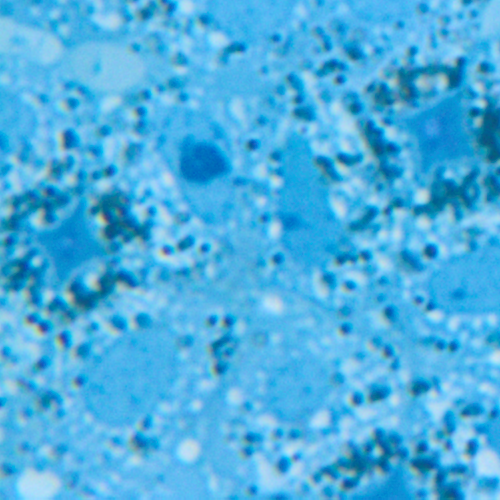

Supplement: S1 File — (ZIP) [file pone.0216281.s001.zip › vacuolas - horisontal eye section photos/black/B_100x_1-5.tif]

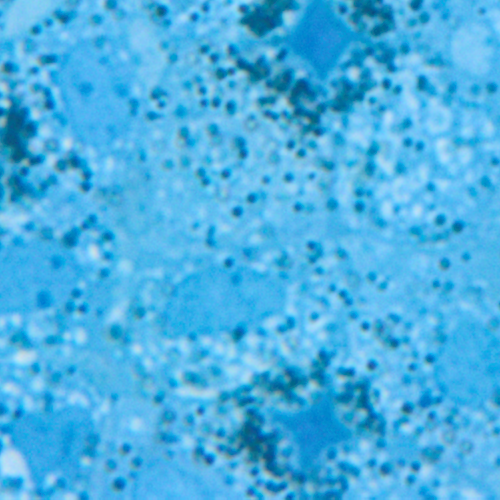

Supplement: S1 File — (ZIP) [file pone.0216281.s001.zip › vacuolas - horisontal eye section photos/black/B_100x_1-6.tif]

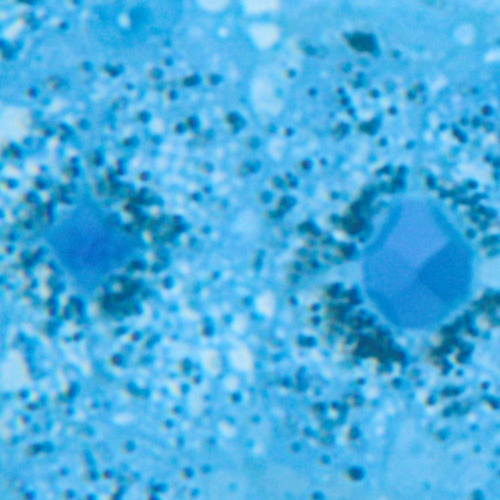

Supplement: S1 File — (ZIP) [file pone.0216281.s001.zip › vacuolas - horisontal eye section photos/black/B_100x_2-1.tif]

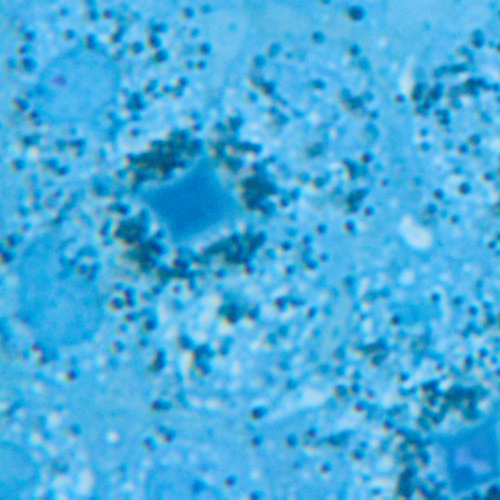

Supplement: S1 File — (ZIP) [file pone.0216281.s001.zip › vacuolas - horisontal eye section photos/black/B_100x_2-2.tif]

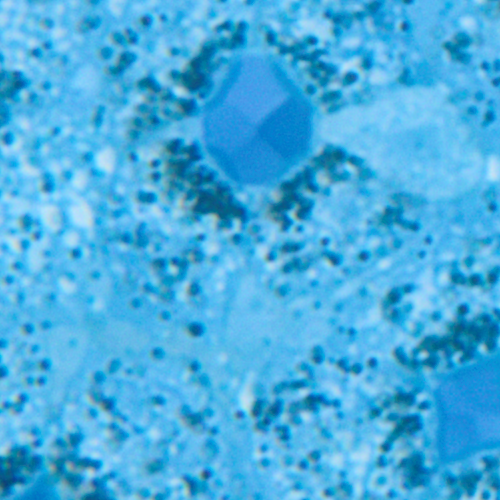

Supplement: S1 File — (ZIP) [file pone.0216281.s001.zip › vacuolas - horisontal eye section photos/black/B_100x_2-3.tif]

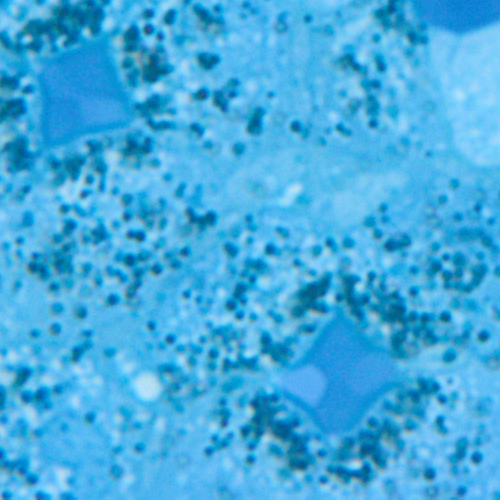

Supplement: S1 File — (ZIP) [file pone.0216281.s001.zip › vacuolas - horisontal eye section photos/black/B_100x_2-4.tif]

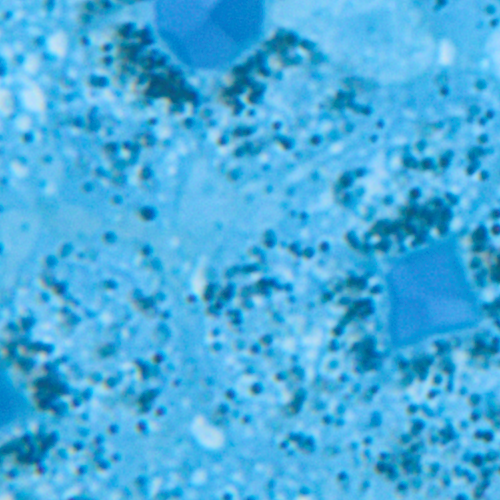

Supplement: S1 File — (ZIP) [file pone.0216281.s001.zip › vacuolas - horisontal eye section photos/black/B_100x_2-5.tif]

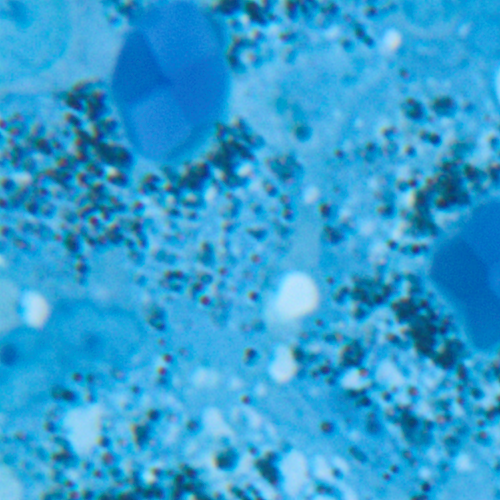

Supplement: S1 File — (ZIP) [file pone.0216281.s001.zip › vacuolas - horisontal eye section photos/black/B_100x_3-1.tif]

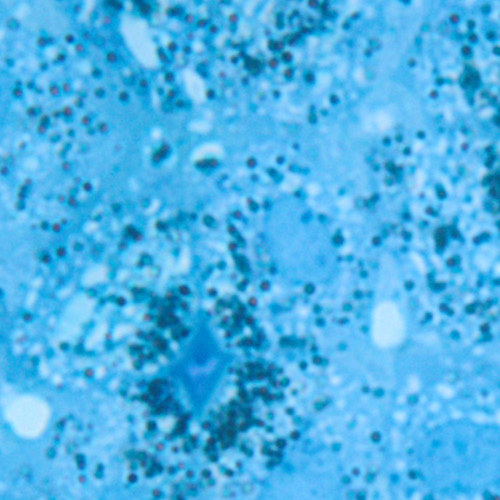

Supplement: S1 File — (ZIP) [file pone.0216281.s001.zip › vacuolas - horisontal eye section photos/black/B_100x_3-2.tif]

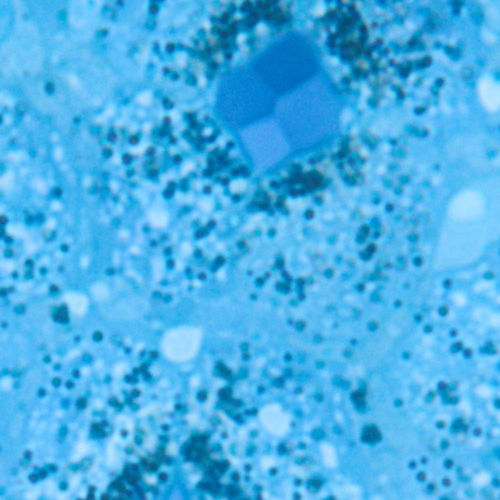

Supplement: S1 File — (ZIP) [file pone.0216281.s001.zip › vacuolas - horisontal eye section photos/black/B_100x_3-3.tif]

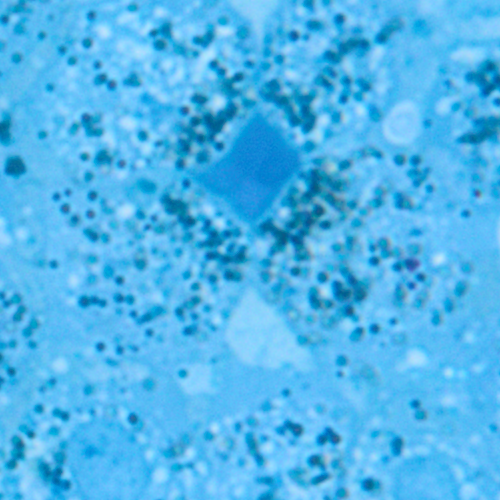

Supplement: S1 File — (ZIP) [file pone.0216281.s001.zip › vacuolas - horisontal eye section photos/black/B_100x_3-4.tif]

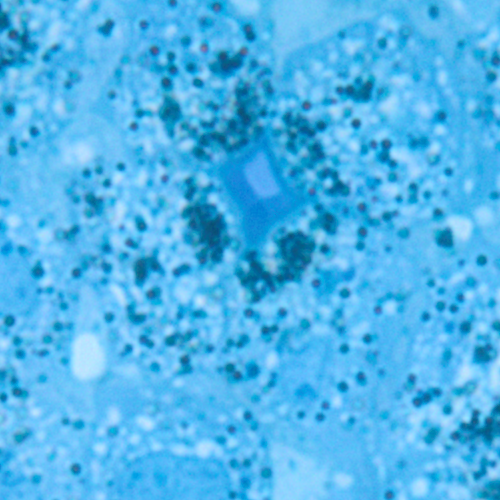

Supplement: S1 File — (ZIP) [file pone.0216281.s001.zip › vacuolas - horisontal eye section photos/black/B_100x_3-5.tif]

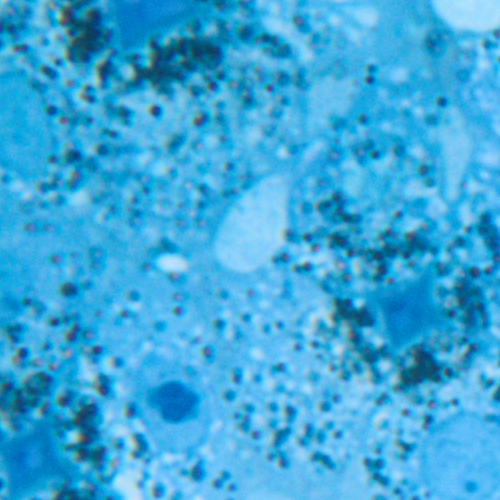

Supplement: S1 File — (ZIP) [file pone.0216281.s001.zip › vacuolas - horisontal eye section photos/black/B_100x_3-6.tif]

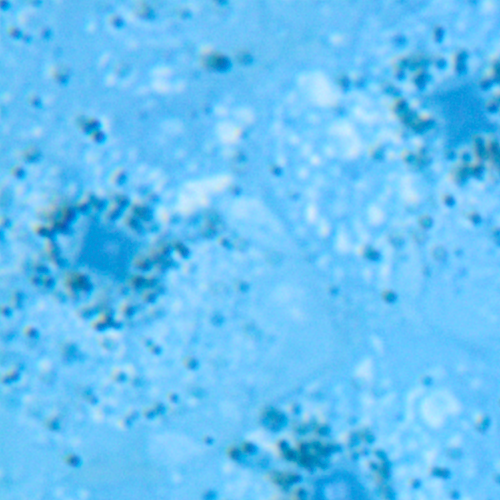

Supplement: S1 File — (ZIP) [file pone.0216281.s001.zip › vacuolas - horisontal eye section photos/black/B_100x_4-1.tif]

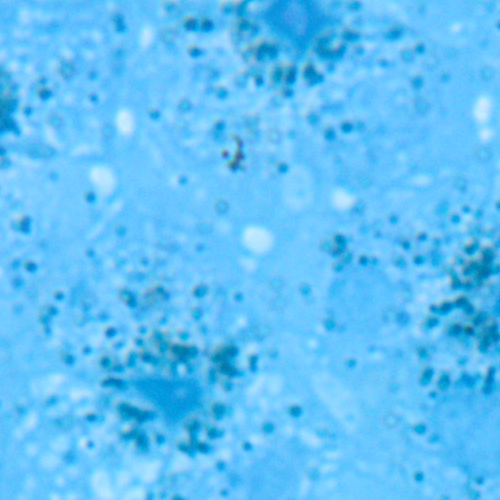

Supplement: S1 File — (ZIP) [file pone.0216281.s001.zip › vacuolas - horisontal eye section photos/black/B_100x_4-2.tif]

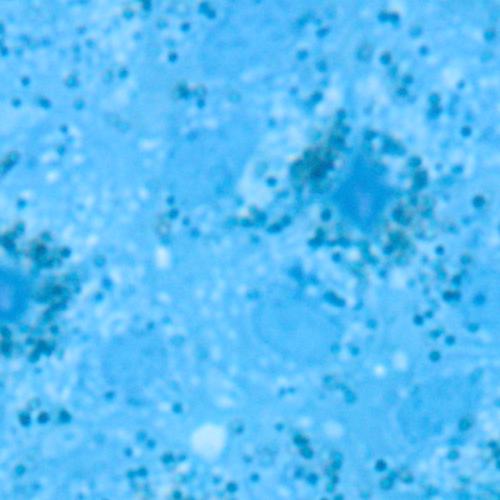

Supplement: S1 File — (ZIP) [file pone.0216281.s001.zip › vacuolas - horisontal eye section photos/black/B_100x_4-3.tif]

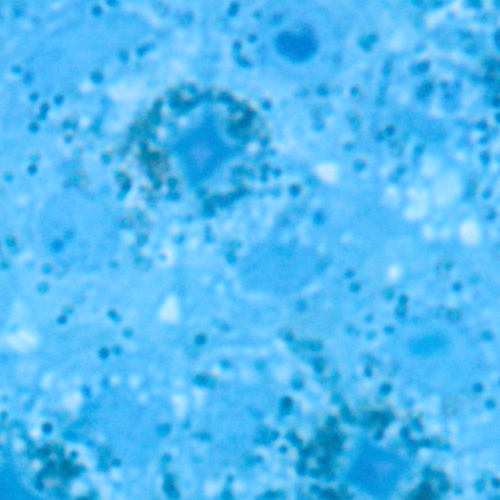

Supplement: S1 File — (ZIP) [file pone.0216281.s001.zip › vacuolas - horisontal eye section photos/black/B_100x_4-4.tif]

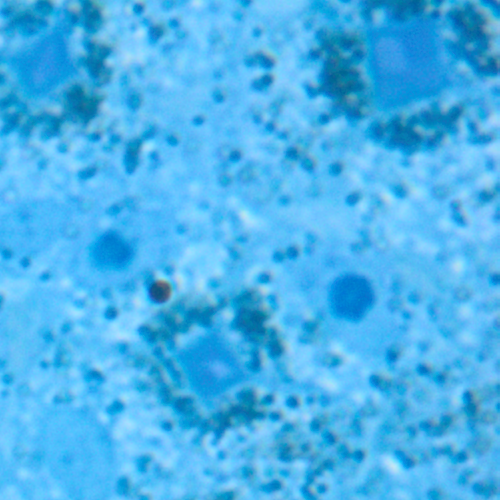

Supplement: S1 File — (ZIP) [file pone.0216281.s001.zip › vacuolas - horisontal eye section photos/black/B_100x_4-5.tif]

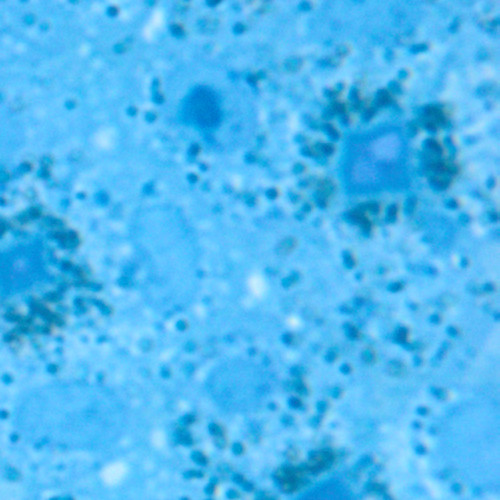

Supplement: S1 File — (ZIP) [file pone.0216281.s001.zip › vacuolas - horisontal eye section photos/black/B_100x_4-6.tif]

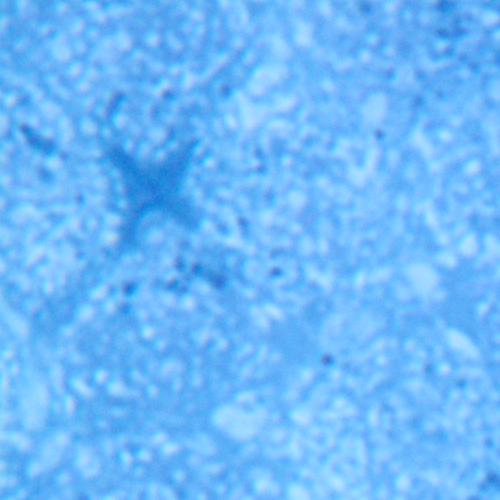

Supplement: S1 File — (ZIP) [file pone.0216281.s001.zip › vacuolas - horisontal eye section photos/white/W_100x_1-1.tif]

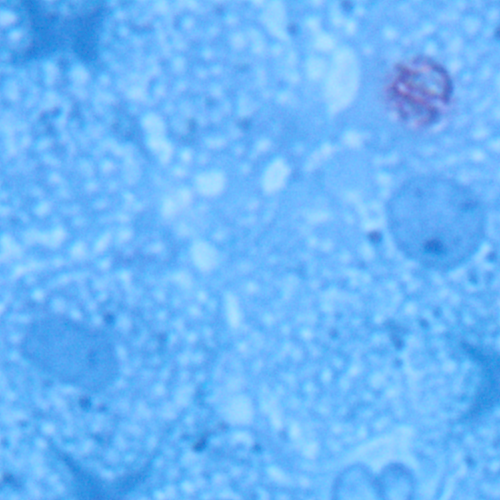

Supplement: S1 File — (ZIP) [file pone.0216281.s001.zip › vacuolas - horisontal eye section photos/white/W_100x_1-2.tif]

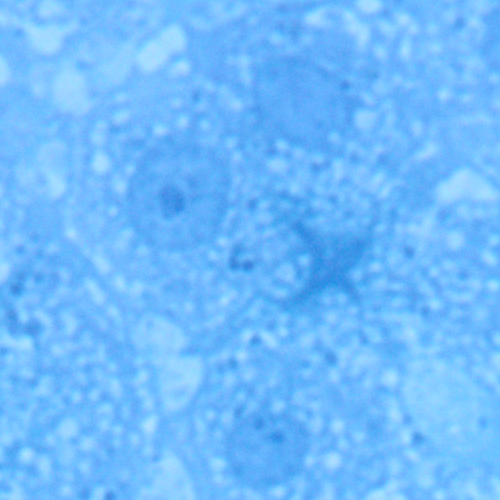

Supplement: S1 File — (ZIP) [file pone.0216281.s001.zip › vacuolas - horisontal eye section photos/white/W_100x_1-3.tif]

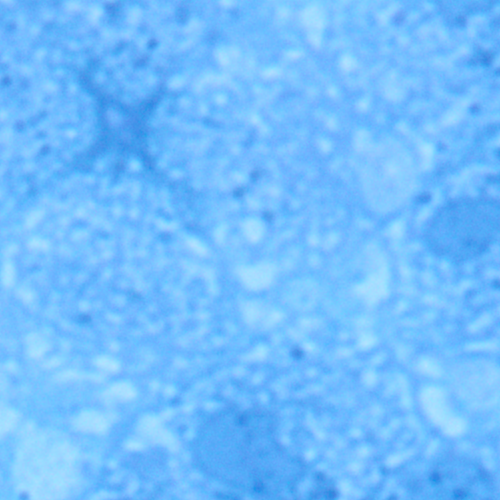

Supplement: S1 File — (ZIP) [file pone.0216281.s001.zip › vacuolas - horisontal eye section photos/white/W_100x_1-4.tif]

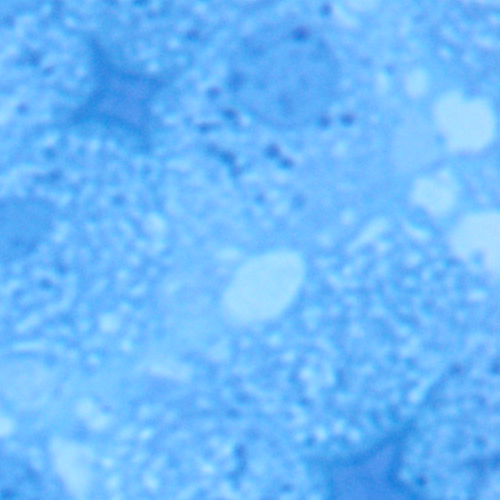

Supplement: S1 File — (ZIP) [file pone.0216281.s001.zip › vacuolas - horisontal eye section photos/white/W_100x_1-5.tif]

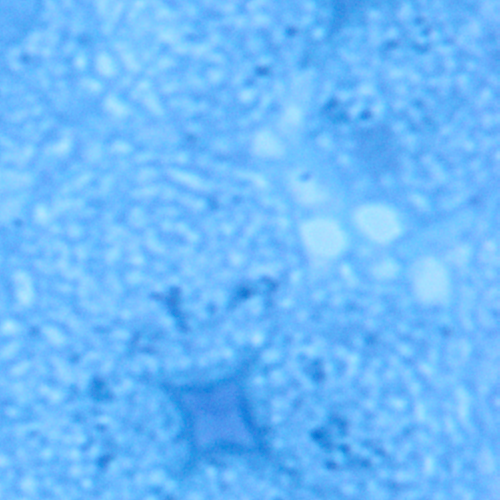

Supplement: S1 File — (ZIP) [file pone.0216281.s001.zip › vacuolas - horisontal eye section photos/white/W_100x_1-6.tif]

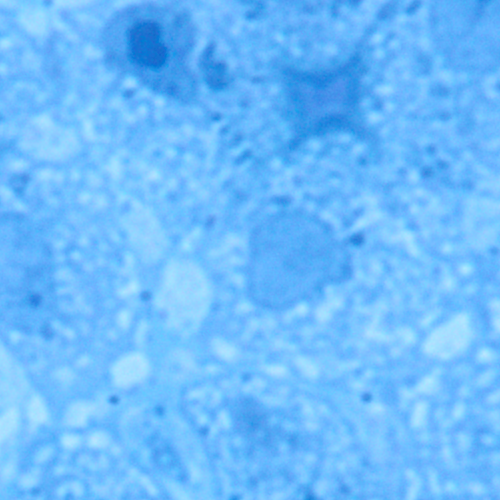

Supplement: S1 File — (ZIP) [file pone.0216281.s001.zip › vacuolas - horisontal eye section photos/white/W_100x_2-1.tif]

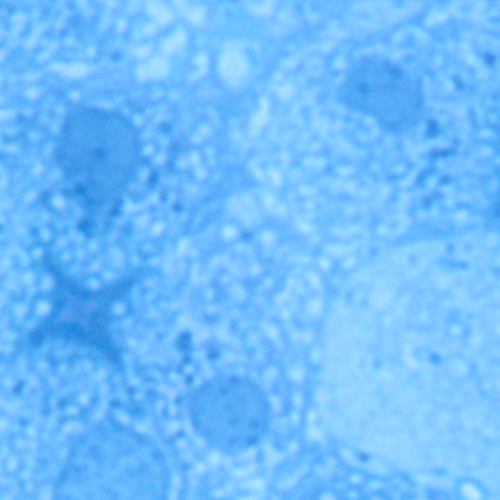

Supplement: S1 File — (ZIP) [file pone.0216281.s001.zip › vacuolas - horisontal eye section photos/white/W_100x_2-2.tif]

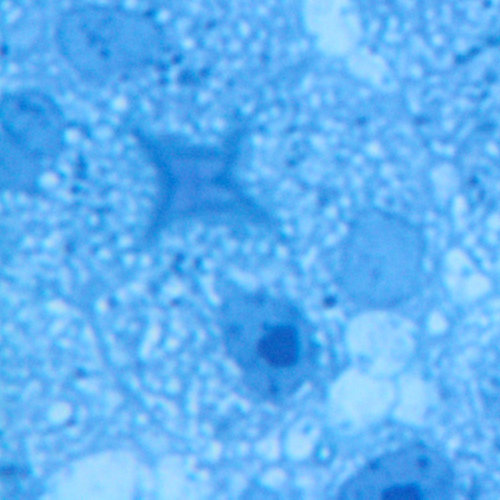

Supplement: S1 File — (ZIP) [file pone.0216281.s001.zip › vacuolas - horisontal eye section photos/white/W_100x_2-3.tif]

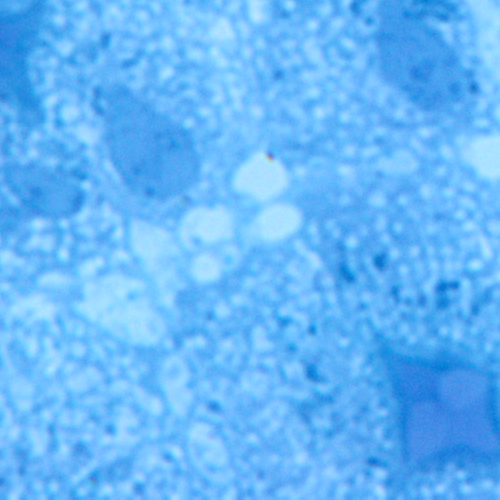

Supplement: S1 File — (ZIP) [file pone.0216281.s001.zip › vacuolas - horisontal eye section photos/white/W_100x_2-4.tif]

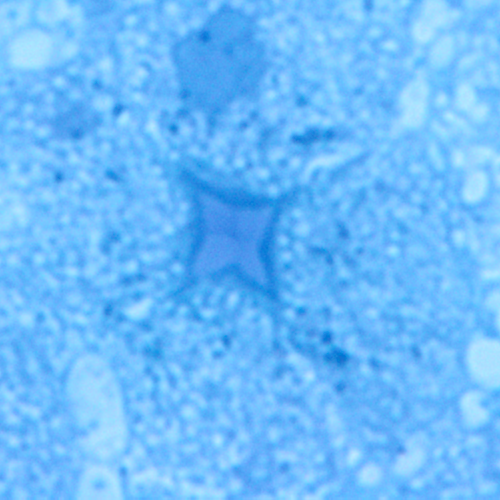

Supplement: S1 File — (ZIP) [file pone.0216281.s001.zip › vacuolas - horisontal eye section photos/white/W_100x_2-5.tif]

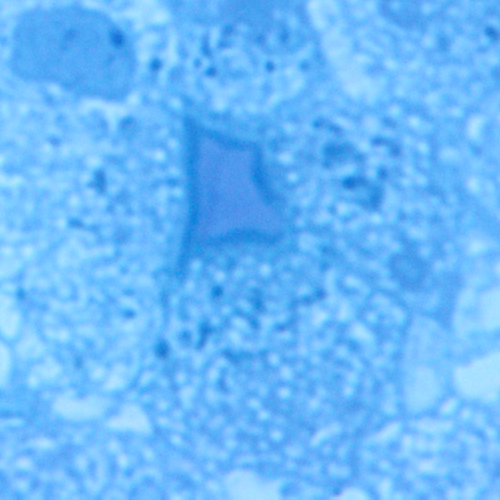

Supplement: S1 File — (ZIP) [file pone.0216281.s001.zip › vacuolas - horisontal eye section photos/white/W_100x_2-6.tif]

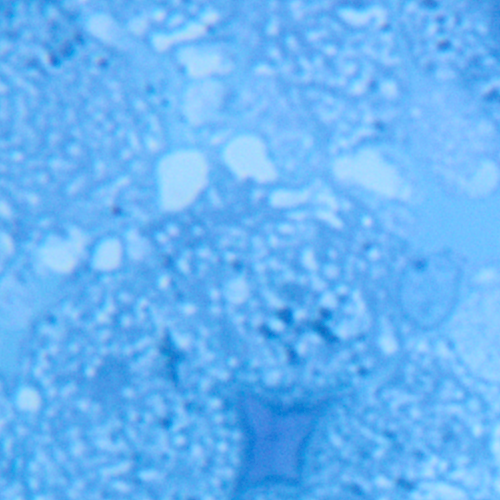

Supplement: S1 File — (ZIP) [file pone.0216281.s001.zip › vacuolas - horisontal eye section photos/white/W_100x_3-1.tif]

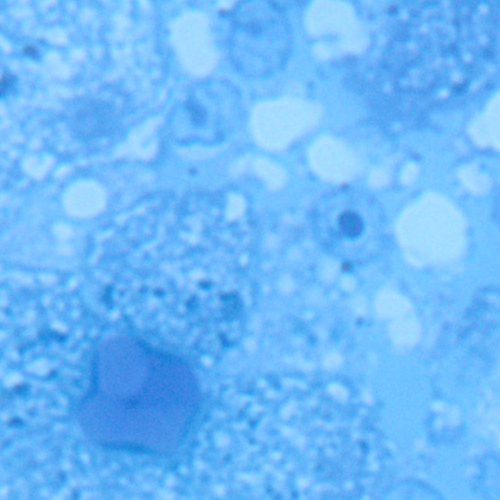

Supplement: S1 File — (ZIP) [file pone.0216281.s001.zip › vacuolas - horisontal eye section photos/white/W_100x_3-2.tif]

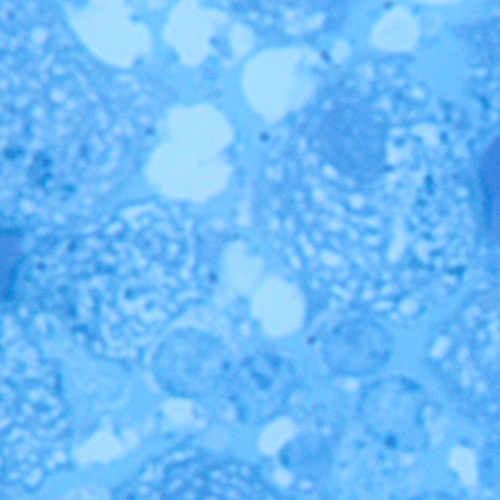

Supplement: S1 File — (ZIP) [file pone.0216281.s001.zip › vacuolas - horisontal eye section photos/white/W_100x_3-3.tif]

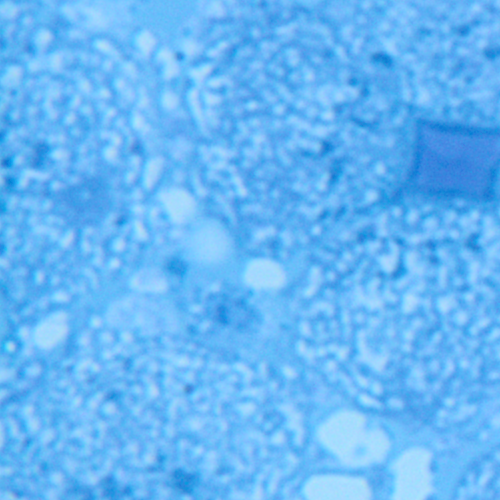

Supplement: S1 File — (ZIP) [file pone.0216281.s001.zip › vacuolas - horisontal eye section photos/white/W_100x_3-4.tif]

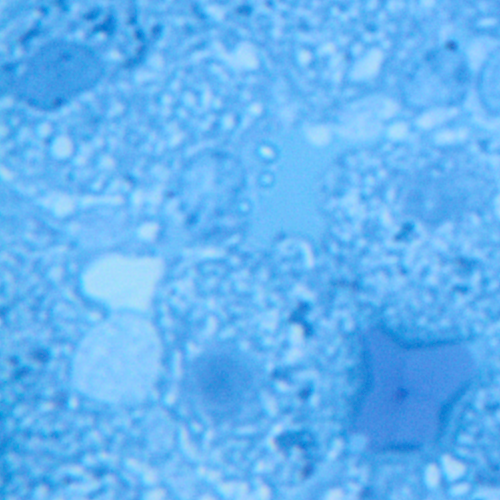

Supplement: S1 File — (ZIP) [file pone.0216281.s001.zip › vacuolas - horisontal eye section photos/white/W_100x_3-5.tif]

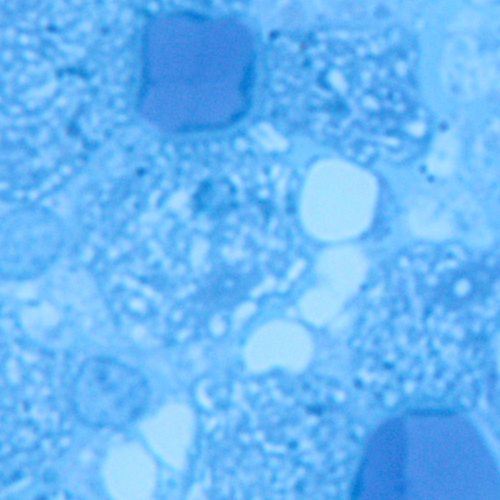

Supplement: S1 File — (ZIP) [file pone.0216281.s001.zip › vacuolas - horisontal eye section photos/white/W_100x_3-6.tif]

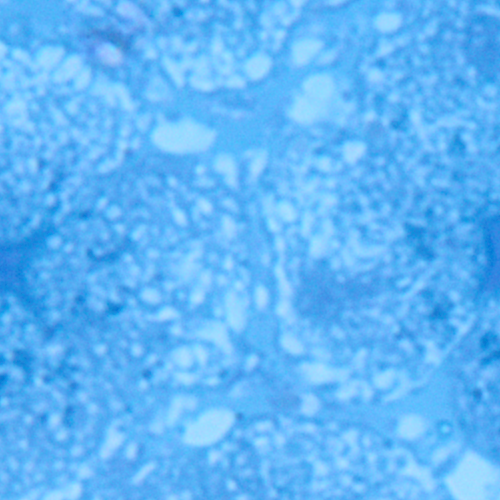

Supplement: S1 File — (ZIP) [file pone.0216281.s001.zip › vacuolas - horisontal eye section photos/white/W_100x_4-1.tif]

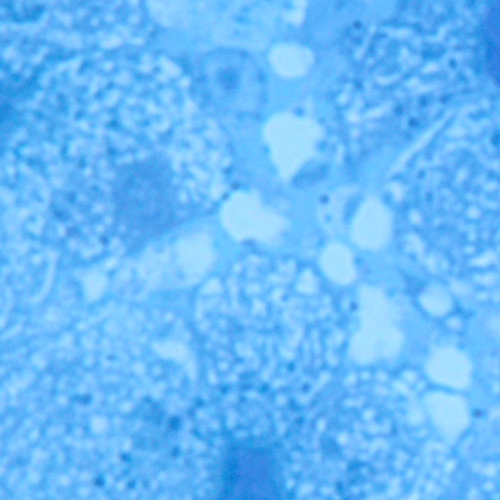

Supplement: S1 File — (ZIP) [file pone.0216281.s001.zip › vacuolas - horisontal eye section photos/white/W_100x_4-2.tif]

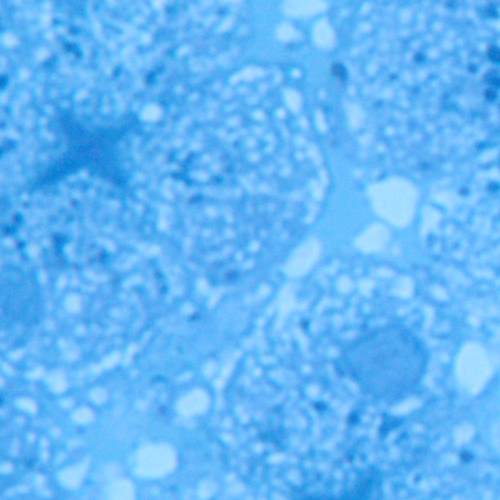

Supplement: S1 File — (ZIP) [file pone.0216281.s001.zip › vacuolas - horisontal eye section photos/white/W_100x_4-3.tif]

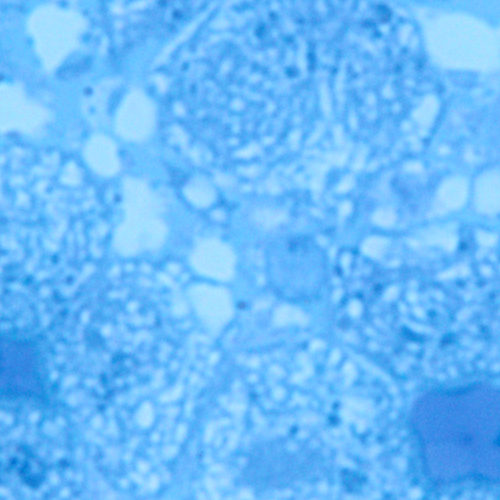

Supplement: S1 File — (ZIP) [file pone.0216281.s001.zip › vacuolas - horisontal eye section photos/white/W_100x_4-4.tif]

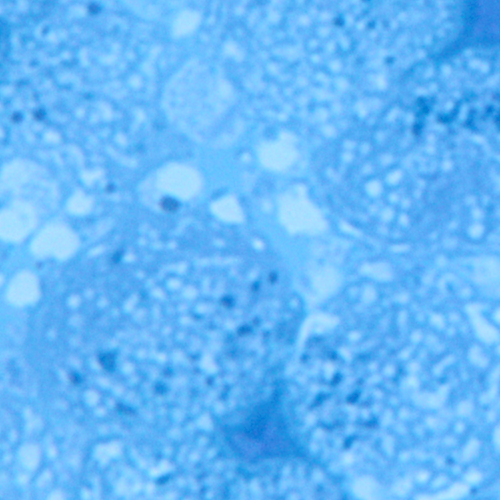

Supplement: S1 File — (ZIP) [file pone.0216281.s001.zip › vacuolas - horisontal eye section photos/white/W_100x_4-5.tif]

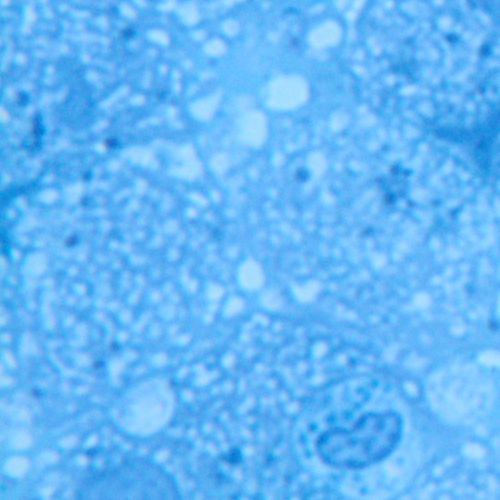

Supplement: S1 File — (ZIP) [file pone.0216281.s001.zip › vacuolas - horisontal eye section photos/white/W_100x_4-6.tif]

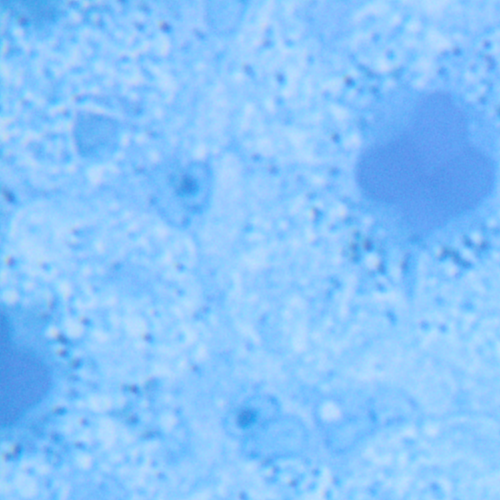

Supplement: S1 File — (ZIP) [file pone.0216281.s001.zip › vacuolas - horisontal eye section photos/yellow/Y_100x_2-1.tif]

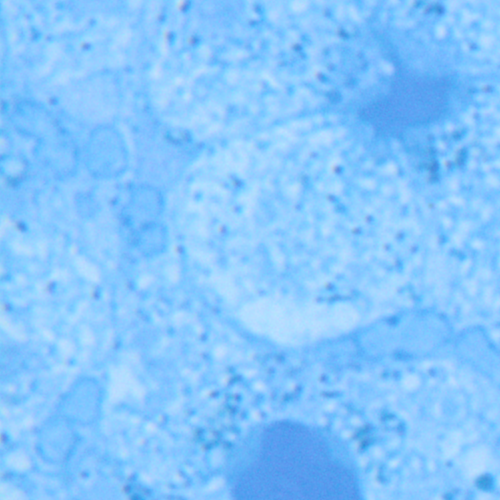

Supplement: S1 File — (ZIP) [file pone.0216281.s001.zip › vacuolas - horisontal eye section photos/yellow/Y_100x_2-2.tif]

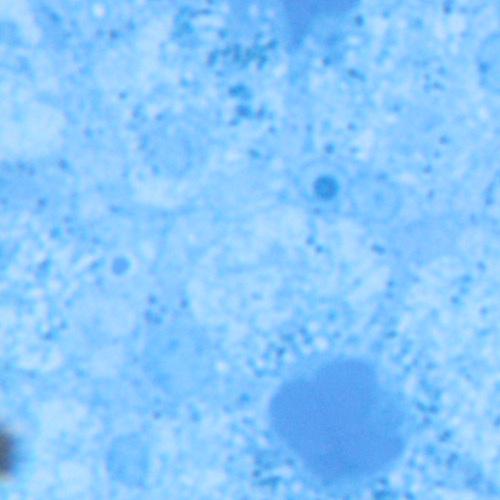

Supplement: S1 File — (ZIP) [file pone.0216281.s001.zip › vacuolas - horisontal eye section photos/yellow/Y_100x_2-3.tif]

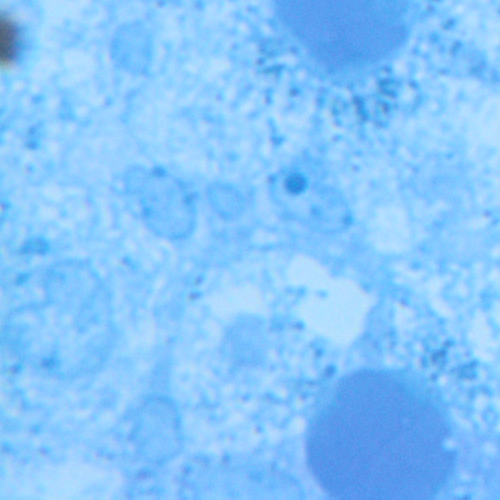

Supplement: S1 File — (ZIP) [file pone.0216281.s001.zip › vacuolas - horisontal eye section photos/yellow/Y_100x_2-4.tif]

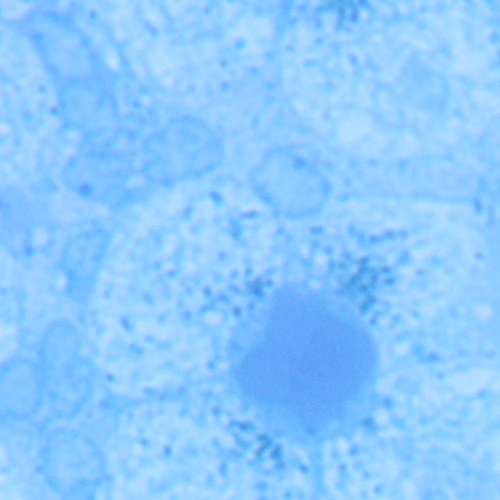

Supplement: S1 File — (ZIP) [file pone.0216281.s001.zip › vacuolas - horisontal eye section photos/yellow/Y_100x_2-5.tif]

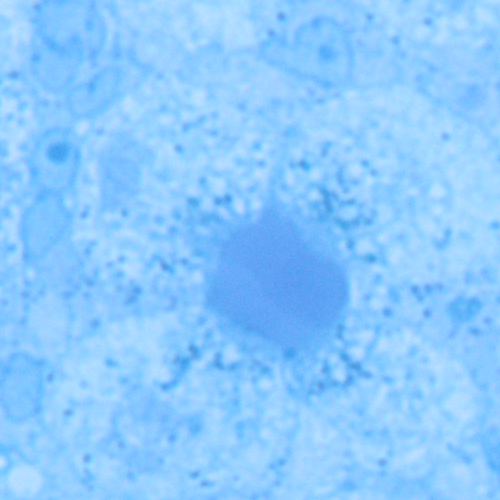

Supplement: S1 File — (ZIP) [file pone.0216281.s001.zip › vacuolas - horisontal eye section photos/yellow/Y_100x_2-6.tif]

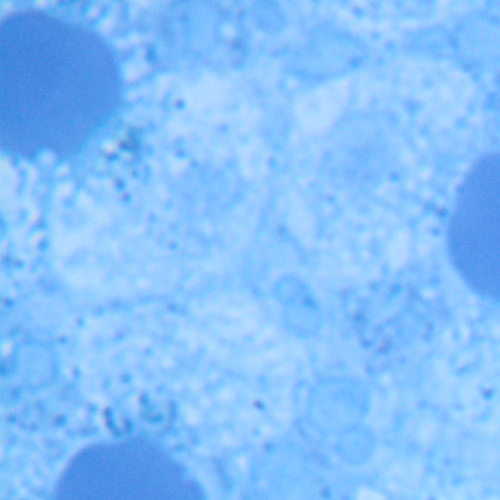

Supplement: S1 File — (ZIP) [file pone.0216281.s001.zip › vacuolas - horisontal eye section photos/yellow/Y_100x_3-1.tif]

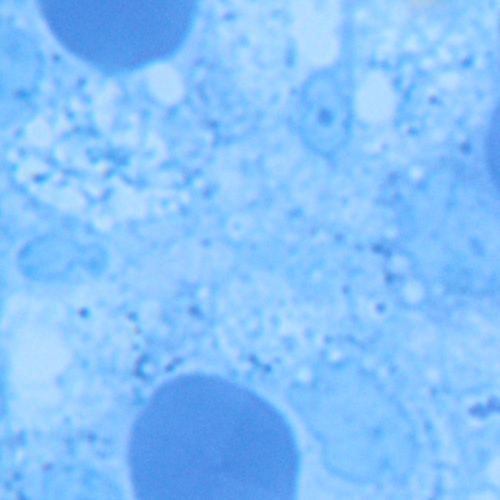

Supplement: S1 File — (ZIP) [file pone.0216281.s001.zip › vacuolas - horisontal eye section photos/yellow/Y_100x_3-2.tif]

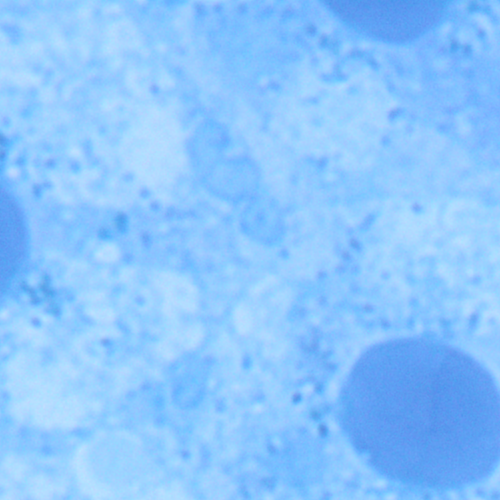

Supplement: S1 File — (ZIP) [file pone.0216281.s001.zip › vacuolas - horisontal eye section photos/yellow/Y_100x_3-3.tif]

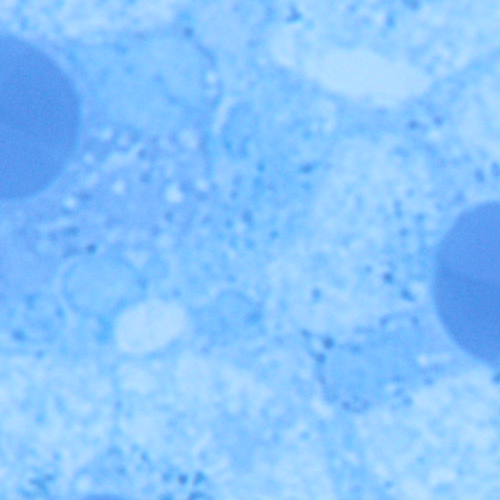

Supplement: S1 File — (ZIP) [file pone.0216281.s001.zip › vacuolas - horisontal eye section photos/yellow/Y_100x_3-4.tif]

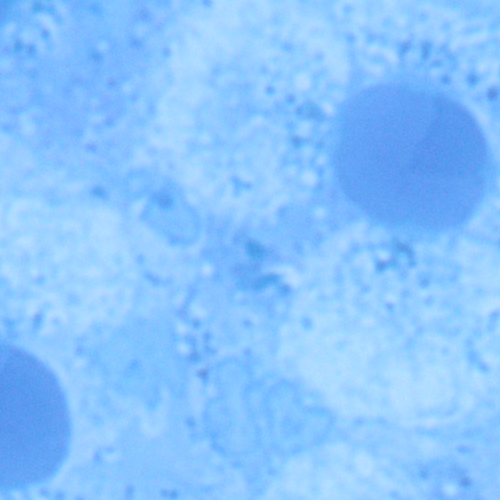

Supplement: S1 File — (ZIP) [file pone.0216281.s001.zip › vacuolas - horisontal eye section photos/yellow/Y_100x_3-5.tif]

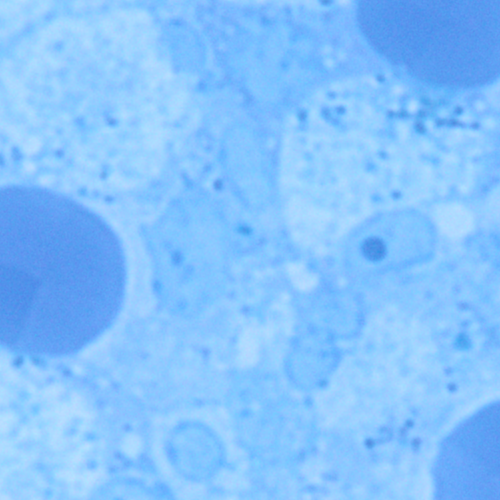

Supplement: S1 File — (ZIP) [file pone.0216281.s001.zip › vacuolas - horisontal eye section photos/yellow/Y_100x_3-6.tif]

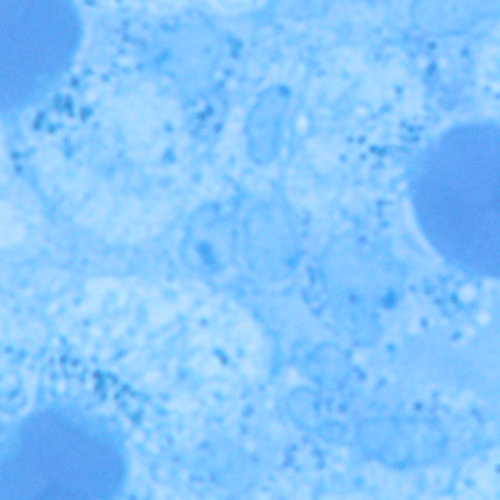

Supplement: S1 File — (ZIP) [file pone.0216281.s001.zip › vacuolas - horisontal eye section photos/yellow/Y_100x_4-1.tif]

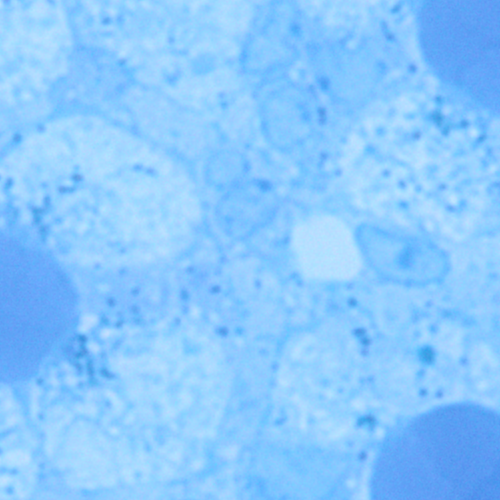

Supplement: S1 File — (ZIP) [file pone.0216281.s001.zip › vacuolas - horisontal eye section photos/yellow/Y_100x_4-2.tif]

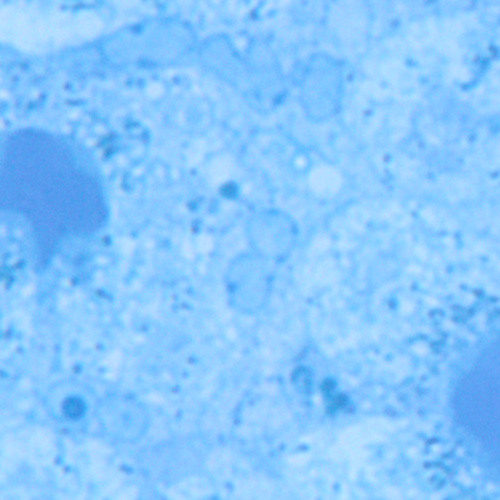

Supplement: S1 File — (ZIP) [file pone.0216281.s001.zip › vacuolas - horisontal eye section photos/yellow/Y_100x_4-3.tif]

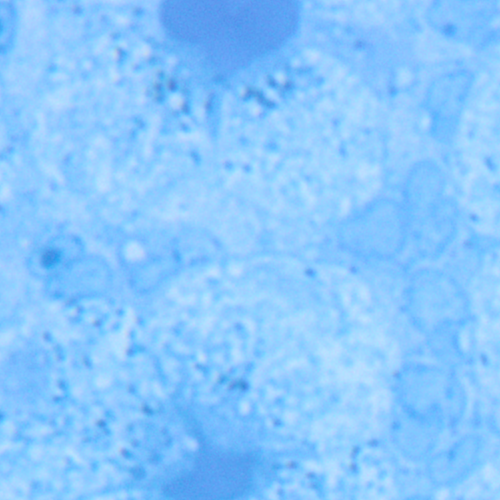

Supplement: S1 File — (ZIP) [file pone.0216281.s001.zip › vacuolas - horisontal eye section photos/yellow/Y_100x_4-4.tif]

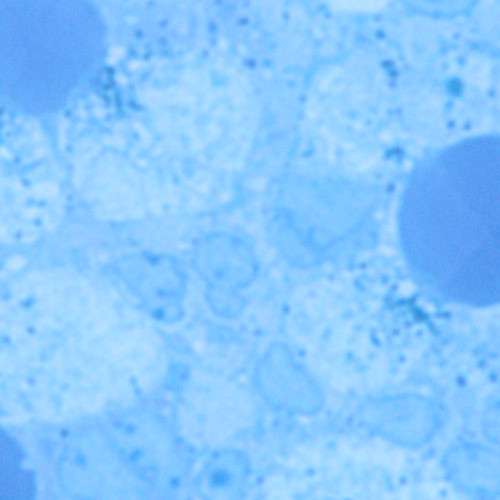

Supplement: S1 File — (ZIP) [file pone.0216281.s001.zip › vacuolas - horisontal eye section photos/yellow/Y_100x_4-5.tif]

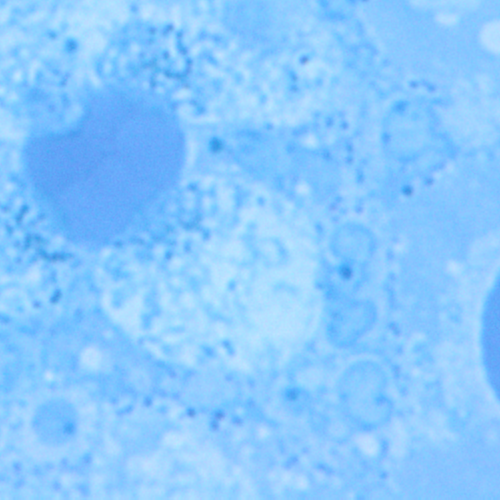

Supplement: S1 File — (ZIP) [file pone.0216281.s001.zip › vacuolas - horisontal eye section photos/yellow/Y_100x_4-6.tif]

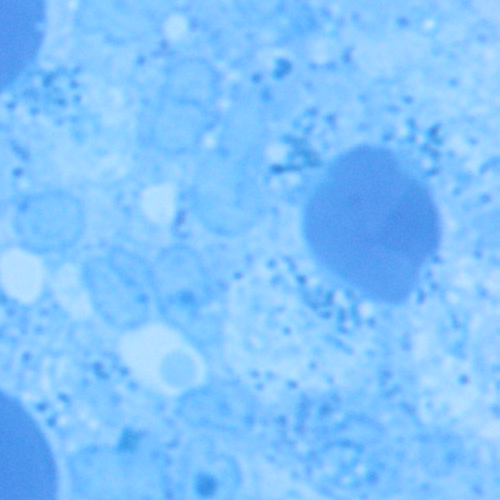

Supplement: S1 File — (ZIP) [file pone.0216281.s001.zip › vacuolas - horisontal eye section photos/yellow/Y_100x_5-1.tif]

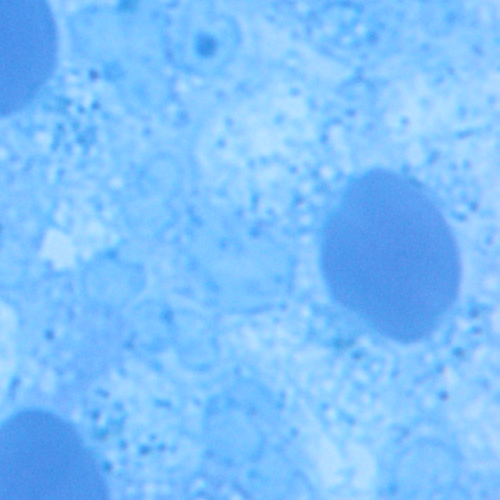

Supplement: S1 File — (ZIP) [file pone.0216281.s001.zip › vacuolas - horisontal eye section photos/yellow/Y_100x_5-2.tif]

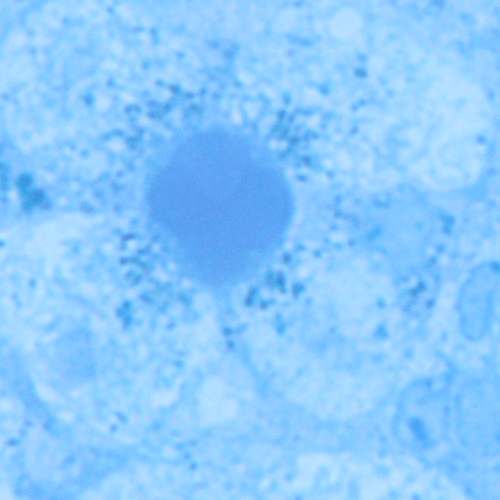

Supplement: S1 File — (ZIP) [file pone.0216281.s001.zip › vacuolas - horisontal eye section photos/yellow/Y_100x_5-3.tif]

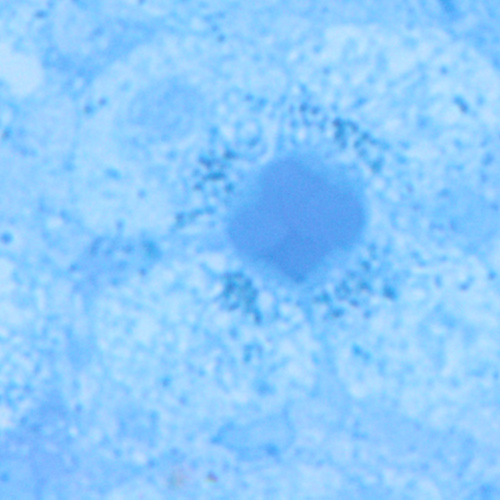

Supplement: S1 File — (ZIP) [file pone.0216281.s001.zip › vacuolas - horisontal eye section photos/yellow/Y_100x_5-4.tif]

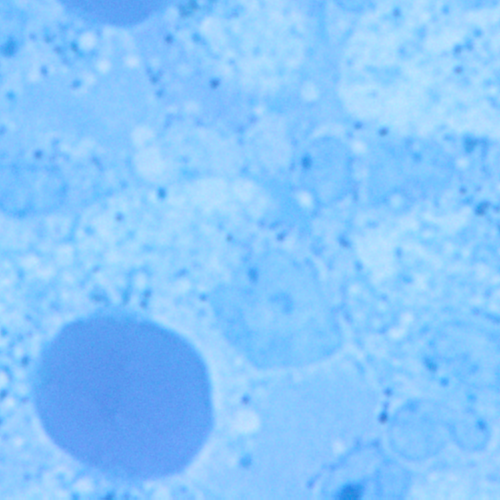

Supplement: S1 File — (ZIP) [file pone.0216281.s001.zip › vacuolas - horisontal eye section photos/yellow/Y_100x_5-5.tif]

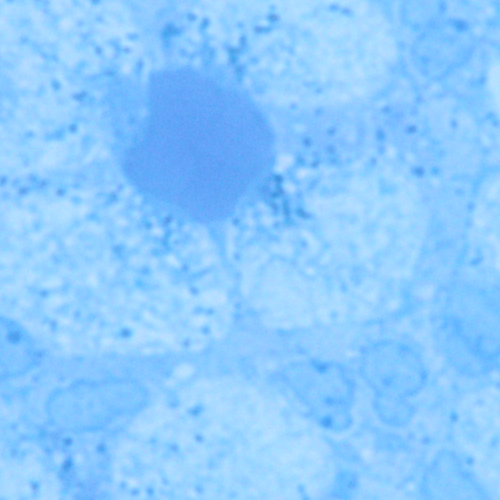

Supplement: S1 File — (ZIP) [file pone.0216281.s001.zip › vacuolas - horisontal eye section photos/yellow/Y_100x_5-6.tif]
